# Cannabidiol attenuates epileptic phenotype and increases survival in a mouse model of developmental and epileptic encephalopathy type 1

**DOI:** 10.1111/epi.18522

**Published:** 2025-07-03

**Authors:** Lucia Verrillo, Fabio Arturo Iannotti, Denise Drongitis, Katiuscia Martinello, Loredana Poeta, Adriano Barra, Gaetano Terrone, Sergio Fucile, Vincenzo Di Marzo, Maria Giuseppina Miano

**Affiliations:** ^1^ Institute of Genetics and Biophysics “Adriano Buzzati‐Traverso,” National Research Council of Italy (CNR) Naples Italy; ^2^ Institute of Biomolecular Chemistry, National Research Council of Italy Pozzuoli, Naples Italy; ^3^ Istituto Neurologico Mediterraneo Pozzilli (Neuromed) ‐ Scientific Institute for Research , Hospitalization and Healthcare (IRCCS) Pozzilli (Isernia) Italy; ^4^ Department of Human Sciences Society and Health University of Cassino and Southern Lazio Cassino (Frosinone) Italy; ^5^ Department of Translational Medicine, Child Neurology, and Psychiatry University of Naples “Federico II” Naples Italy; ^6^ Department of Physiology and Pharmacology Sapienza University of Rome Rome Italy; ^7^ Joint International Unit Between the CNR of Italy and Université Laval (Quebec, Canada) on Chemical and Biomolecular Research on the Microbiome and Its Impact on Metabolic Health and Nutrition Quebec Canada

**Keywords:** Arx polyalanine mice, cannabidiol, neurexin splicing switches, pharmacoresistant seizure, *Pparg*, TRPV1

## Abstract

**Objective:**

Developmental and epileptic encephalopathy type 1 (DEE1) is a rare drug‐resistant pediatric epilepsy caused by trinucleotide repeat expansions in the X‐linked *ARX* gene, leading to elongation of the first polyalanine tract. It presents with early onset tonic seizures or spasms, developmental and cognition delay, and high risk of premature mortality. We evaluated the therapeutic potential of highly purified cannabidiol (CBD) in *Arx* ^(GCG)7/Y^ mice, a genetic DEE1 model that replicates key features of the human condition.

**Methods:**

*Arx* ^(GCG)7/Y^ mice received daily intraperitoneal CBD (100 mg/kg) for 7 days. The epileptic phenotype was evaluated via video monitoring and a scoring matrix. In *Arx*‐DEE1 male cortex, real‐time polymerase chain reaction and Western blotting assessed CBD effects on proinflammatory and neuronal markers. Microglial morphology was analyzed by Iba1 immunostaining and Sholl analysis. In vitro patch‐clamp recordings tested CBD activity on *Arx* ^(GCG)7/Y^ cortical neurons.

**Results:**

CBD reduced the severity and frequency of spontaneous recurrent seizures and significantly extended the lifespan of epileptic mice. In mutant symptomatic mice, CBD activated peroxisome *Pparg* expression and the concurrent desensitization/inactivation of TRPV1 channels. Additionally, CBD counteracted the dysregulated expression of the proinflammatory genes *Ptgs2, Mmp9, Il12, Cd68, Ccl2,* and *Irf3,* while also restoring normal microglial morphology. Further molecular analysis demonstrated that CBD effectively offsets normal alternative splicing for the presynaptic receptor genes *Nrnx1* and *Nrnx3*. Consistent with this, CBD rescued proper *Nrnx1* splicing in mutant cortical neurons after K^+^‐induced depolarization. Finally, we found that CBD reduced neuronal excitability by inducing hyperpolarization, raising the action potential threshold, and reducing the frequency and mean charge of inhibitory postsynaptic currents and the mean charge of excitatory postsynaptic currents.

**Significance:**

These findings represent the first preclinical evidence of CBD efficacy in a murine model of genetic DEE1, identifying CBD‐sensitive downstream targets and paving the way to further exploration of CBD effects in this disease for future clinical consideration.

**Key points**

- Mice with GCG trinucleotide expansion in *Arx* gene (*Arx* ^(GCG)7/Y^) present severe seizures and high risk of mortality.
- Treatment with CBD in young *Arx*‐mutated mice reduces frequency and severity of spontaneous recurrent seizures and increases overall survival.
- CBD treatment dampens neuroinflammation, reducing the expression of proinflammatory genes and correcting microglia morphology.
- The anti‐ictogenic effects of CBD may be mediated by decreasing neuronal excitability.

## 1 INTRODUCTION

Developmental and epileptic encephalopathies (DEEs) represent a clinically and genetically heterogeneous group of rare neurodevelopmental disorders (NDDs) characterized by early onset of drug‐resistant epilepsy, typical electroencephalographic (EEG) patterns, developmental delay or regression, particularly after the onset of refractory seizures, and a complex range of comorbidities, ranging from movement disorders to autismlike symptoms.^1^ To date, many DEE genes have been identified, including those encoding transcription factors, ion channels, or proteins involved in neurotransmission.^2^

Expanded runs of consecutive mixed (GCN)n repeats in the first and second polyalanine tract of the Aristaless‐related homeobox gene (*ARX*; Mendelian Inheritance in Man [MIM] 300382) have been identified in male children with a severe form of DEE, termed DEE type 1 (DEE1; MIM 308350), also known as infantile spasm syndrome X‐linked type 1(ISSX1).^3^, ^4^ *ARX* is an X‐chromosome gene that encodes a bifunctional high‐hierarchy homeotic transcription factor with a pivotal role in cortex development.^5^ DEE1 is part of a phenotypic spectrum of *ARX*‐related diseases—generally affecting only male children—including lissencephaly (MIM 300215), Proud syndrome (MIM 300004), DEE1 (MIM 308350), and syndromic (MIM 309510) and nonsyndromic (MIM 300419) intellectual disability.^5^, ^6^ *ARX*‐DEE1 patients typically develop pharmacoresistant infantile epileptic spasms (ES), associated with a characteristic pattern on EEG called hypsarrhythmia, development arrest, and high risk for premature death.^7^, ^8^ Mechanistically, expanded ARX proteins show reduced transactivation activity and impaired DNA binding at specific gene‐regulatory regions, altering the broad ARX‐dependent transcriptional program.^4^, ^9^, ^10^ Given the broad spectrum of ARX functions, which are finely tuned both spatially and temporally with cellular specificity, a complete delineation of the multiple molecular and cellular processes damaged by polyalanine elongations remains a challenging and only partially accomplished task.^11^, ^12^ In this context, our previous research revealed abnormalities in neuronal network formation, translation efficiency, and RNA splicing in the neonatal brains of *Arx* transgenic mice.^12^

Patients with *ARX*‐related DEE may display different epileptic phenotypes, ranging from early infantile epileptic encephalopathy with a suppression–burst pattern on EEG and myoclonic seizures to infantile ES, all characterized by severe pharmacoresistance.^13^, ^14^ The limited effectiveness of current antiseizure medications (ASMs) highlights the persistent demand from clinicians and families for the identification of new therapeutic strategies for this early onset DEE. A promising pharmacological therapy with 17β‐estradiol (E2) has been tested in *Arx* ^(GCG)10+7/Y^ mice harboring the insertion of eight GCG alanine codons in the first *Arx* polyalanine stretch.^15^, ^16^ This is a vital knockin *Arx*‐epileptic male model characterized by spontaneous spasmlike myoclonus in pups and seizures in young animals, in which presymptomatic E2 treatment halts spasmlike myoclonus.^15^, ^16^ Beneficial effects of early E2 treatment were also observed in *Arx* ^(GCG)7/Y^ mice.^17^ This is a different knockin *Arx*‐ epileptic male model harboring the insertion of seven GCG alanine codons in the first polyalanine stretch.^11^, ^15^, ^17^, ^18^, ^19^ *Arx* ^(GCG)7/Y^ young mice exhibit severe spontaneous tonic–clonic seizures—which generally start at postnatal day (PND) 30—and present a high mortality incidence.^11^, ^15^, ^17^, ^18^, ^19^ In this *Arx*‐polyalanine model, E2 treatment ameliorates seizure frequency but has no effect on mortality rate.^17^ All this evidence highlights the urgent need to explore the potential efficacy of new ASMs suitable for these disorders.

In recent years, highly purified cannabidiol (CBD) derived from *Cannabis sativa* was approved by the US Food and Drug Administration and European Medicines Agency for the treatment of seizures associated with Dravet syndrome (DS; MIM 607208)^20^ Lennox–Gastaut syndrome,^21^ and tuberous sclerosis complex^22^ in patients 2 years of age and older. Regarding its mechanism of action, CBD exerts a broad range of effects at the molecular and cellular level, influencing inflammation, pain, excitability, and other physiological and pathophysiological processes including peroxisome proliferator‐activated receptor gamma (PPARγ) receptors and transient receptor potential vanilloid subtype 1 (TRPV1) channels, among others.^23^, ^24^ Although the anticonvulsant action of CBD has been observed in other DEEs, including infantile epileptic spasms syndrome patients, the lack of sufficient studies demonstrating its efficacy has hindered its approval for the treatment of additional DEEs.^25^, ^26^

This study is the first to explore postnatal CBD treatment in epileptic *Arx* ^(GCG)7/Y^ mice, a preclinical model of early onset DEE1. We demonstrate that daily administration of CBD significantly improves the epileptic behavior and extends lifespan in *Arx* ^(GCG)7/Y^ mice. We further reveal unanticipated secondary molecular and cellular changes driven by *Arx*‐polyalanine elongation that CBD effectively reverses. Overall, these findings establish a preclinical framework for the antiseizure potential of CBD in DEE1 and provide scientific rationale for further exploration of the effects of CBD in this disease toward future clinical consideration.

## 2 MATERIALS AND METHODS

All materials and methods are described in Supporting Information.

## 3 RESULTS

### 3.1 CBD reduces seizure frequency in *Arx*‐DEE1 mice

**FIGURE 1:**
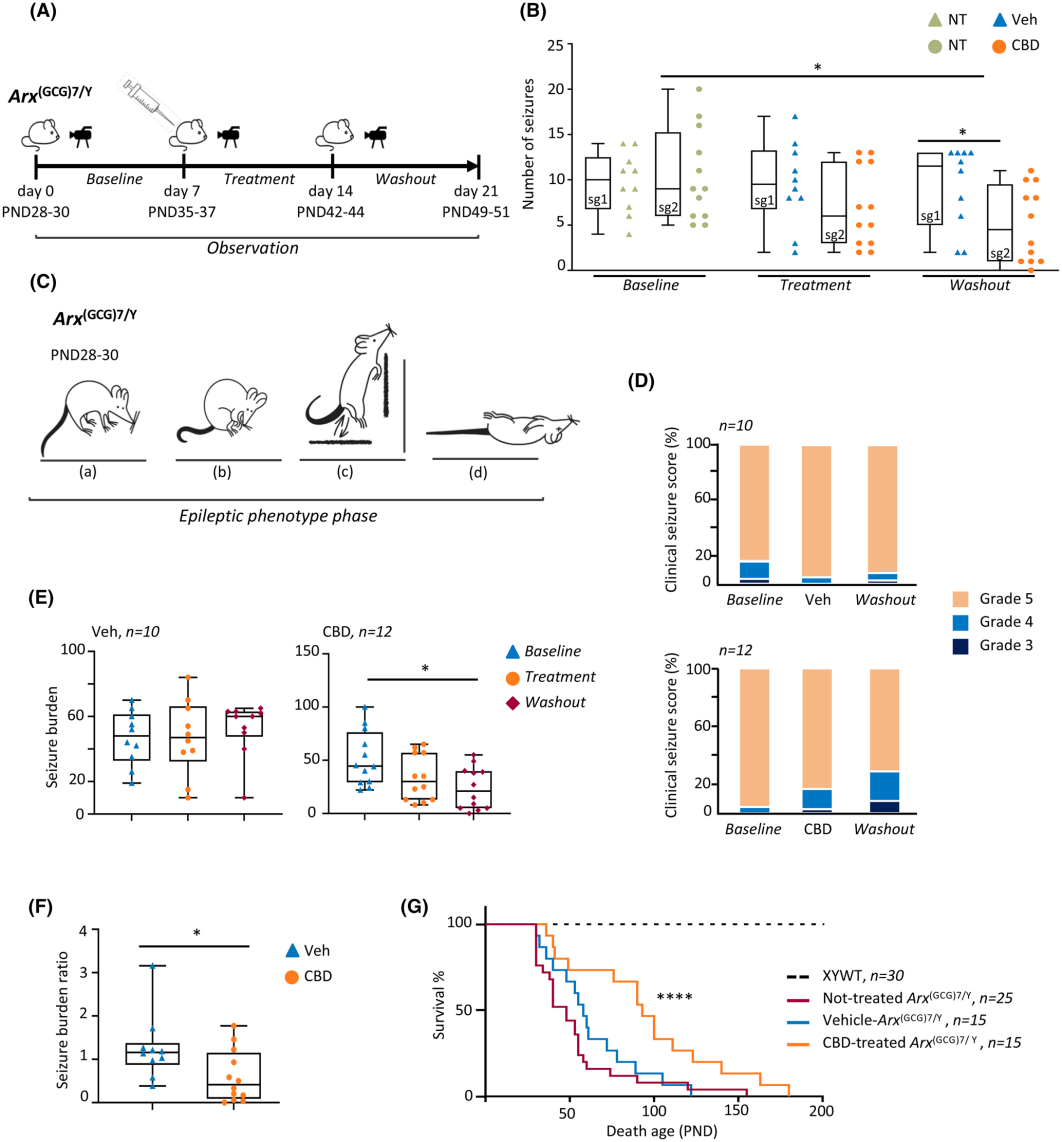
Cannabidiol (CBD) reduces spontaneous seizure frequency and ameliorates epileptic phenotype and survival in epileptic *Arx* ^(GCG)7/Y^ mice. (A) Study timeline for the epileptic *Arx* ^(GCG)7/Y^ animals. Video‐monitoring analysis of mouse behavior during baseline, treatment, and washout phases is shown (see Section 2 for details). PND, postnatal day. (B) Boxplots depicting the median number of seizures per day in study group 1 (sg1) for vehicle (Veh) treatments (*n* = 10) and study group 2 (sg2) for CBD treatments (*n* = 12). One‐way repeated measures analysis of variance (ANOVA) with Holm–Sidak multiple comparisons: *p < .02. Individual data points plotted beside the boxes are the averaged data for individual mice during each phase of the experiment. NT, no treatment. (C) Schematic representation of the epilepsy behavior of *Arx* ^(GCG)7/Y^ animals: (a) head down, (b) stopped and huddled, (c), wild jumping against the wall, (d) sudden death. Credit: E. Castagna. (D) Spine plots depicting the clinical seizure scores as grade 3, 4, or 5. The graphic was based on seizure ratings/animal for each phase of the study (baseline, treatment with vehicle or CBD, and washout). (E) Boxplots depicting seizure burden for each phase of the study (baseline, treatment with vehicle or CBD, and washout; *n* = 12/CBD and *n* = 10/Veh groups). D'Agostino–Pearson test and one‐way repeated measures ANOVA, followed by Tukey multiple comparison test, were applied (*p = .0360). (F) Boxplots depicting seizure burden ratio for each treatment period with vehicle or CBD. Mann Whitney test was applied (*p < .04). Individual data points plotted beside the boxes are the averaged data for individual mice during each phase of the experiment. (G) Kaplan–Meier survival curves in CBD‐treated and vehicle‐treated *Arx* ^(GCG)7/Y^ mice. Significant survival differences of CBD group versus vehicle group were calculated by applying Mantel–Cox log‐rank test (****p < .0001).

To determine the effect of CBD on spontaneous recurrent seizures (SRS) in symptomatic *Arx* ^(GCG)7/Y^ young mice, we assessed seizure frequency and duration before, during, and after 1 week of daily intraperitoneal administration of CBD (100 mg/kg) or vehicle (Veh). The experimental workflow is depicted in Figure 1A, summarizing the treatment strategy including the baseline, treatment, and washout phases. *Arx* ^(GCG)7/Y^ young mice (PND28–30) were assigned in a random and blinded manner to receive Veh (study group 1 [sg1]: Veh‐treated *Arx* ^(GCG)7/Y^, *n* = 10) or CBD (study group 2 [sg2]: CBD‐treated *Arx* ^(GCG)7/Y^, *n* = 12).

Before initiating treatments, the epileptic phenotype was analyzed in both study groups sg1 and sg2 (baseline; Figure 1A). All seizures started with trembling of the limbs and progressed to tonic–clonic convulsions, running fits, and then complete loss of postural control and movement, as previously described.^19^ Seven days of video recording showed that *Arx* ^(GCG)7/Y^ (*n* = 5) experienced frequent SRS (approximately 2 episodes per day) with a median duration of 30 s (Figure S1A and Video S1).

The two experimental groups sg1 Veh‐treated *Arx* ^(GCG)7/Y^ and sg2 CBD‐treated *Arx* ^(GCG)7/Y^ received once‐daily intraperitoneal injections respectively of Veh or CBD for 7 days (PND35–37) and were recorded (treatment); treatments were then discontinued, and mice (PND42–44) were recorded for the next 7 days (washout; Figure 1A). This additional recording time enabled us to assess seizure frequency after drug washout and to address any potential disease‐modifying effects of CBD (Figure 1A). No differences in SRS frequency were detected in Veh‐treated *Arx* ^(GCG)7/Y^ during treatment and after washout phases. On the contrary, we observed a significant reduction in seizure frequency in CBD‐treated *Arx* ^(GCG)7/Y^ during the washout phase in comparison to the baseline and treatment phases (Figure 1B). Specifically, median seizures were 9 (interquartile range [IQR] = 9.25–6, *n* = 125 total seizures) during the baseline period, 6 (IQR = 12–3, *n* = 84 total seizures) during CBD daily treatment, and finally, 4.25 (IQR = 9.5–1, *n* = 61 seizures) during the posttreatment period. Concerning the seizure duration, we found a similar distribution during baseline (29 s, IQR = 40–20), treatment (35 s, IQR = 45–25), and washout phases (35 s, IQR = 40–23) in the two experimental groups CBD‐treated *Arx* ^(GCG)7/Y^ and Veh‐treated *Arx* ^(GCG)7/Y^ (Figure S1B). Given these results, we conclude that CBD reduces the frequency of SRS in *Arx* ^(GCG)7/Y^ mice in a time‐dependent manner, without affecting seizure duration. Importantly, its effects persist, although briefly (for 7 days), after the treatment is discontinued.

### 3.2 CBD reduces seizure severity and extends lifespan of *Arx*‐DEE1 mice

Next, we assessed whether CBD alleviates disease severity in addition to seizure frequency. As summarized in Figure 1C, *Arx* ^(GCG)7/Y^ mice show very severe SRS, with head nodding, forelimb clonus, loss of postural tone, rearing, falling, wild jumping, and finally sudden death, mainly scored as 4 and 5 according to the Racine scale score of severity (Table S1).^27^ Clinical seizure score was evaluated upon behavioral analysis in both experimental groups CBD‐treated *Arx* ^(GCG)7/Y^ and Veh‐treated *Arx* ^(GCG)7/Y^. In the baseline phase, seizures were mostly graded 5 as expected in both experimental groups, and during and after the Veh treatments (Figure 1D, upper panel). During CBD treatments, 3.6% and 15.5% of CBD‐treated *Arx* ^(GCG)7/Y^ were grade 3 and 4, respectively; during post‐CBD treatments, 11.5% and 18% of CBD‐treated *Arx* ^(GCG)7/Y^ were grade 3 and 4, respectively (Figure 1D, bottom panel). Given that both seizure severity and frequency contribute significantly to disease manifestation, we also measured the seizure burden, a comprehensive parameter that accounts for both the severity of the epilepsy phenotype and the frequency of seizures.^28^ We found that the Veh‐treated group showed stable seizure burden median values with no significant differences at baseline (median value = 48), treatment (median value = 47), and washout (median value = 60) phases (Figure 1E). On the contrary, a significantly lower seizure burden was observed in the CBD group in the washout (median value = 21) compared to the baseline (median value = 44.5; Figure 1E). In line with the beneficial effects of CBD, we also observed a significantly lower seizure burden ratio in CBD‐treated *Arx* ^(GCG)7/Y^ mice with respect to Veh‐treated *Arx* ^(GCG)7/Y^ mice (Figure 1F).

To determine whether CBD administration affects mortality risk, we compared the survival rates of CBD‐treated mutant mice with those of untreated and Veh‐treated groups, excluding animals in the untreated group that died before weaning. Notably, *Arx* ^(GCG)7/Y^ mice typically experience early mortality, often as a result of severe epileptic fits.^19^, ^29^ Consistent with previous studies, we found that approximately 38% of *Arx* ^(GCG)7/Y^ mice died before weaning (PND28), and the majority of those that survived weaning died within 3 months of age (PND90; Figure 1G, Figure S1C). Upon treatments, we observed a significant increase in survival of CBD‐treated *Arx* ^(GCG)7/Y^ with a median survival of 93 days compared to the Veh‐treated *Arx* ^(GCG)7/Y^ with a median survival of 58 days (log‐rank test, *p* < .0001), suggesting a strong protective CBD effect (Figure 1G). No significant differences in weight changes were observed between the Veh‐treated and CBD‐treated groups (Figure S1D). Collectively, these results demonstrate the antiepileptic effects of CBD in improving epileptic outcomes and reducing mortality in *Arx* ^(GCG)7/Y^ mice, highlighting the potential of CBD as a primary treatment option following a DEE1 diagnosis.

### 3.3 CBD offsets the defective expression levels of the cannabinoid‐related targets PPARγ and TRPV1

Recent investigations have increasingly elucidated the role of cannabinoid‐related targets in mediating the anticonvulsant effects of CBD. Notably, CBD anti‐ictogenic properties have been associated with its interaction with specific molecular pathways, particularly involving PPARγ and the TRPV1 channels. Remarkably, the upregulation of PPARγ was found to correlate with the anticonvulsant action of CBD, suggesting a possible contribution of this receptor in epilepsy management.^30^ Additionally, TRPV1 channels, which are cation‐permeant, have been identified as contributors to neuronal excitability. These channels are often found to be overexpressed in the brains of various rodent models, ex vivo systems, and postmortem analyses of patients with epilepsy. By desensitizing TRPV1 channels, CBD could reduce neurotransmission and neuronal excitability, thereby exerting its anticonvulsant effects. Similar outcomes have been observed with TRPV1 agonists, such as capsaicin, and antagonists such as capsazepine, or TRPV1 knockout, further supporting this mechanism.^31^

**FIGURE 2:**
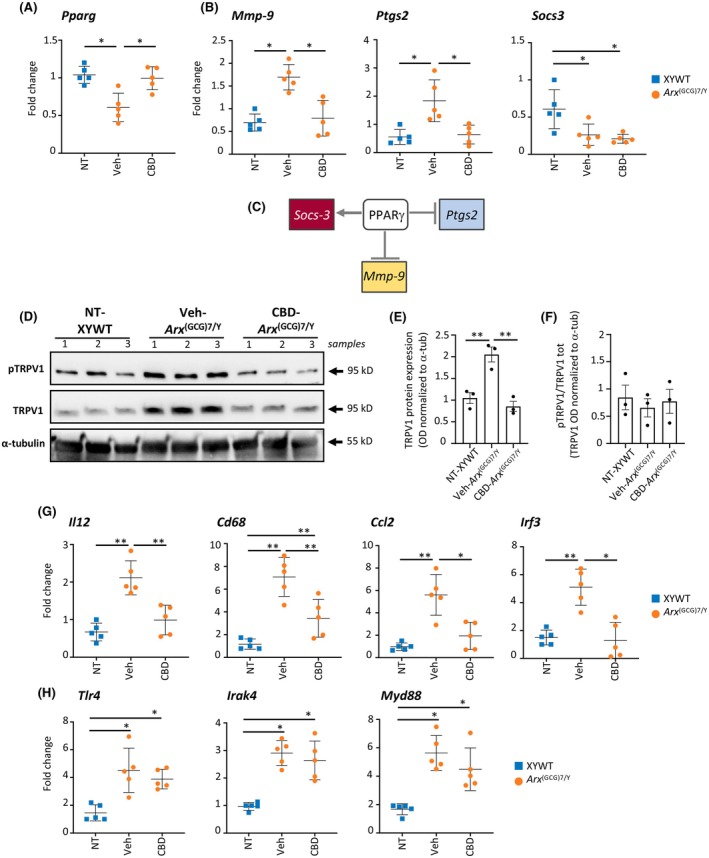
Analysis of the cannabinoid targets *Pparg* and TRPV1 and of neuroinflammatory markers that are cannabidiol (CBD)‐responsive in the *Arx* ^(GCG)7/Y^ cortex. (A, B) mRNA expression of *Pparg*, *Mmp9*, *Ptgs2*, and *Socs3* in the cortex of XYWT, vehicle (Veh)‐treated *Arx* ^(GCG)7/Y^, and CBD‐treated *Arx* ^(GCG)7/Y^ mice measured by quantitative real‐time polymerase chain reaction (qRT‐PCR). Transcript levels were quantified using the 2^−ΔΔCt^ method normalizing to 18S rRNA as the internal control (mean ± SEM of three replicates from five mice for experimental group). CBD, CBD treatment; NT, no treatment; Veh, vehicle treatment. Differences were assessed using two‐way analysis of variance (ANOVA) with Tukey multiple comparisons: *p < .05. (C) Transcriptional axis of PPARγ effector genes. (D) Western blot analysis of phosphorylated TRPV1 (pTRPV1) and TRPV1 expression in the cortex of NT‐XYWT, Veh‐treated *Arx* ^(GCG)7/Y^, and CBD‐treated *Arx* ^(GCG)7/Y^ mice. Each band was normalized against the corresponding α‐tubulin band used as loading control. (E) Bar chart with data points showing the quantification of TRPV1 levels normalized to α‐tubulin (α‐tub). (F) Bar chart with data points showing the quantification of pTRPV1 levels normalized to total TRPV1. Differences were assessed using one‐way ANOVA: **p < .01. Data are from three separate samples. (G, H) mRNA expression of *Il12*, *Cd68*, *Ccl2, Irf3*, *Tlr4*, *Myd88*, and *Irak4* in the cortex of XYWT, *Arx* ^(GCG)7/Y^‐Veh, and *Arx* ^(GCG)7/Y^‐CBD mice measured by qRT‐PCR. Transcript levels were quantified using the 2^−ΔΔCt^ method normalizing to 18S rRNA as the internal control (mean ± SEM of three replicates from five mice for experimental group). Differences were assessed using two‐way ANOVA with Tukey multiple comparisons: *p < .05, **< .005. Optical Density (OD) refers to the measure of intensity of a protein band on the Western blot.

In light of these findings, we moved to explore whether the effects of CBD we observed in DEE1 mice were dependent on PPARγ and TRPV1. To do this, *Arx* ^(GCG)7/Y^ young mice were treated by daily intraperitoneal administration with CBD (100 mg/kg, *n* = 5) or Veh (*n* = 5) for 7 days, at the same age as the behavioral study (PND35–37; Figure 1). We first performed quantitative real‐time polymerase chain reaction (qRT‐PCR) of *Pparg* mRNA levels in the cortex isolated from *Arx* ^(GCG)7/Y^‐Veh and *Arx* ^(GCG)7/Y^‐CBD mice and sex‐ and age‐matched wild type male controls (XYWT). We found a striking reduction in *Pparg* transcript levels in the epileptic cortex of Veh‐treated *Arx* ^(GCG)7/Y^ mice compared to XYWT mice (Figure 2A). CBD completely restores *Pparg* transcript levels to match those of control mice, suggesting that *Pparg* downregulation may be an intrinsic mechanism contributing to the DEE1 pathophysiology (Figure 2A). Notably, the *Pparg* gene is a confirmed ARX target,^32^ containing a 5′‐TAATTA‐3′ ARX binding motif^4^ located in a conserved noncoding element of its promoter region (Figure S2).^33^ Given the established antineuroinflammation properties of *PPARG*,^34^ we next assessed whether CBD could also exert a favorable effect on three PPARγ target genes involved in inflammation: *Mmp*‐*9* and *Ptgs2*, which are negative PPARγ targets and mediate proinflammatory responses,^34^ and *Socs3*, a gene transcriptionally activated by PPAR*γ* that suppresses cytokine signaling (Figure 2B,C).^35^ Consistent with the *Pparg* levels observed in Veh‐treated *Arx* ^(GCG)7/Y^ and CBD‐treated *Arx* ^(GCG)7/Y^ mice, the transcript levels of *Mmp*‐*9* and *Ptgs2* were found respectively increased in Veh‐treated mutant cortex and decreased in CBD‐treated mutant cortex (Figure 2B). In contrast, *Socs3* was significantly downregulated in the epileptic cortex of both Veh‐treated *Arx* ^(GCG)7/Y^ and CBD‐treated *Arx* ^(GCG)7/Y^ mice (Figure 2B), indicating that CBD specifically targets the transcriptional axis of *Pparg* with *Mmp*‐*9* and *Ptgs2*, while leaving *Socs3* expression unchanged.

By Western blot analysis, we then measured the protein levels of TRPV1 and phosphoTRPV1 (Ser502 residue) in the cortex isolated from Veh‐treated *Arx* ^(GCG)7/Y^ and CBD‐treated *Arx* ^(GCG)7/Y^ mice and sex‐ and age‐matched controls (XYWT) using specific antibodies (Figure 2D–F). Consistent with previous research showing that *Ptgs2* expression increases along with the upregulation of TRPV1,^36^ we found significantly higher levels of TRPV1 and phosphorylated TRPV1 (pTRPV1) in the cortex of epileptic Veh‐treated *Arx* ^(GCG)7/Y^ mice compared to XYWT mice (Figure 2D–F). In contrast, in the cortex of CBD‐treated *Arx* ^(GCG)7/Y^ mice, the levels of these proteins were comparable to those in control animals (Figure 2D–F). Altogether, these findings suggest that CBD, also in the epileptic model we analyzed, exerts its beneficial effects through the promotion of PPARγ expression and concomitant inactivation of TRPV1 channels. Furthermore, they highlight a newly identified role of these CBD‐sensitive markers as key factors in the onset of the epileptic phenotype in DEE1 mice.

### 3.4 CBD suppresses the mRNA levels of the proinflammatory genes *Il12, Cd68, Ccl2*, and *Irf3*

Given the extensive anti‐inflammatory activity of CBD, we next focused on analyzing potential anti‐inflammatory targets that could underlie the beneficial effects observed on the epileptic phenotype of DEE1 mice. To this end, we analyzed the transcript levels of the inflammasome mediators *Il1β, Il6, Il12, Il10, Tnfα, Cd68, Ccl2*, and *Ccl5*, which are upregulated in pilocarpine‐ and kainic acid‐induced epilepsy models.^37^, ^38^ Furthermore, the mRNA levels of *Irf3, Tlr4, Myd88*, and *Irak4* genes—encoding innate immune system markers essential for maintaining neuronal excitation/inhibition balance and implicated in epilepsy—were examined.^39^, ^40^ qRT‐PCR analysis was carried out in the cortex isolated from *Arx* ^(GCG)7/Y^ young mice treated by daily intraperitoneal administration with CBD (100 mg/kg, *n* = 5) or Veh (*n* = 5) for 7 days, at the same age as the behavioral study (PND35–37; Figure 1). We found that the expression levels of *Il12, Cd68, Ccl2*, and *Irf3* were significantly elevated in the *Arx* ^(GCG)7/Y^ cortex, and these increases were notably reduced with CBD treatment (Figure 2G). In contrast, CBD had no effect on the upregulation of *Tlr4*, *Irak4*, or *Myd88* (Figure 2H), and no significant differences were observed in the levels of *Tnfα*, *Il10*, *Il1β*, *Il6*, and *Ccl5* between mutant and control mice (Figure S3). Overall, alongside the observed reversal of the *Pparg*‐ *Ptgs2*‐*Mmp9* axis and TRPV1 protein levels, these results strongly support the hypothesis that CBD exerts its therapeutic effects on the DEE1 phenotype by modulating a specific set of pro‐ and anti‐inflammatory molecules.

### 3.5 CBD alleviates morphological changes in cortical microglia

**FIGURE 3:**
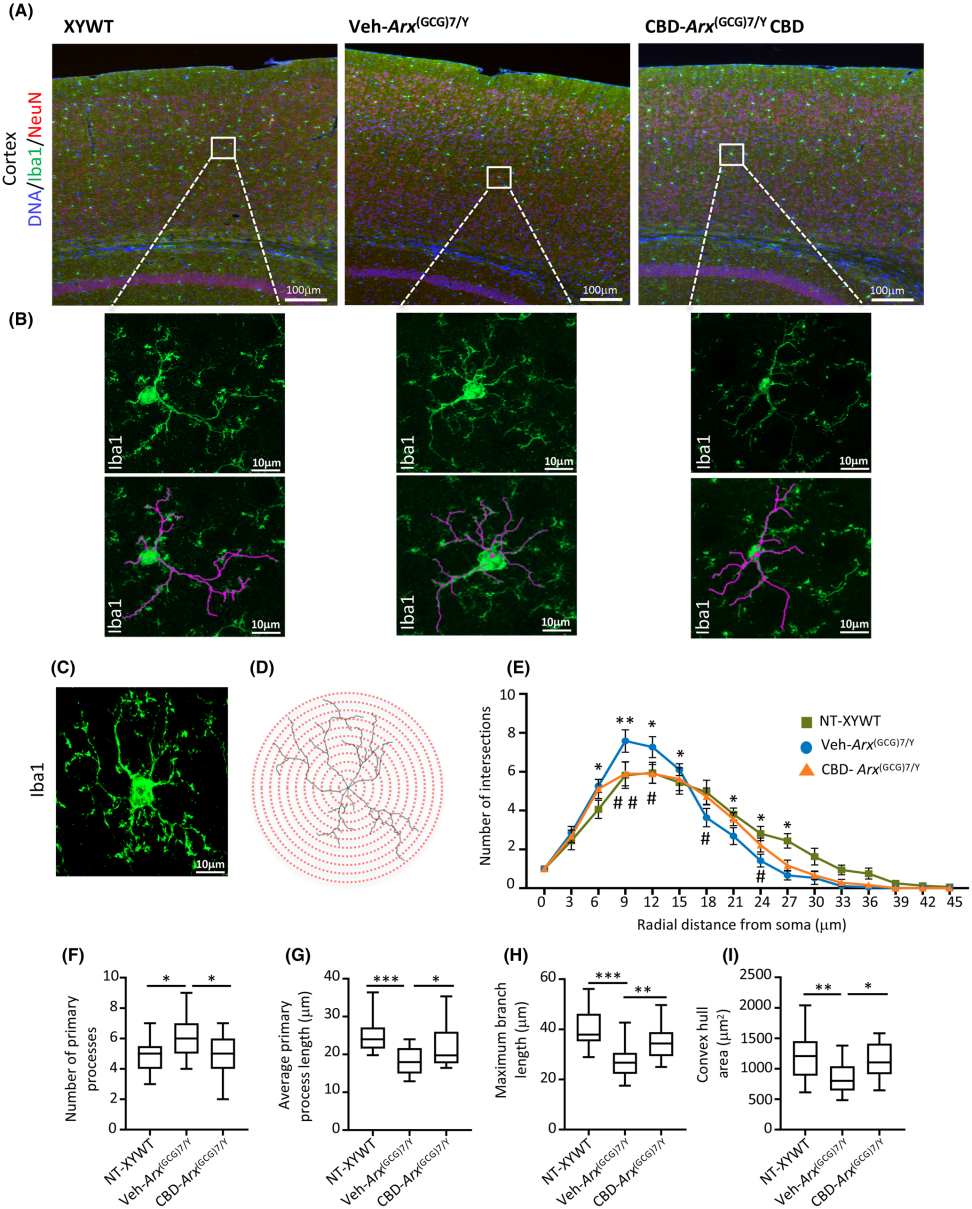
Analysis of microglia morphology in the cortex of XYWT, vehicle (Veh)‐treated *Arx* ^(GCG)7/Y^, and cannabidiol (CBD)‐treated *Arx* ^(GCG)7/Y^. (A, B) Representative confocal micrographs images of Iba1‐positive cells in the cortical subfield of control, Veh‐treated *Arx* ^(GCG)7/Y^, and CBD‐treated *Arx* ^(GCG)7/Y^ mice. Scale bars = 100 μm (A) and 10 μm (B). (C, D) Representative images of cortical Iba1‐positive microglial cell converted to a maximum intensity Z‐stack projection and corresponding binary image created by thresholding with concentric radii imposed upon cells at 3‐μm intervals for the Sholl analysis. Scale bar = 10 μm. (E) Sholl analysis of the number of intersections of microglial processes with a series of concentric circles centered on the soma of each cell in control, Veh‐treated *Arx* ^(GCG)7/Y^, and CBD‐treated *Arx* ^(GCG)7/Y^ mice. The branching profile of microglia from Veh‐treated *Arx* ^(GCG)7/Y^ mice—compared to the XYWT and *Arx* ^(GCG)7/Y^ CBD profiles—is shifted upward near the soma and leftward in the distal region of the soma. CBD, CBD treatment; NT, no treatment; Veh, Veh treatment. Differences were assessed using two‐way analysis of variance (ANOVA) with Tukey multiple comparisons: XYWT versus Veh‐treated *Arx* ^(GCG)7/Y^ *p < .05, **p < .005; Veh‐treated *Arx* ^(GCG)7/Y^ versus CBD‐treated *Arx* ^(GCG)7/Y^ #*p* < .05, ##*p* < .005. (F–I) Analysis of primary processes, average of primary process length, maximum of branch length, and convex hull area. Cells numbered *n* = 18 for XYWT, *n* = 19 for Veh‐treated *Arx* ^(GCG)7/Y^, and *n* = 18 for CBD‐treated *Arx* ^(GCG)7/Y^. Differences were assessed using two‐way ANOVA with Tukey multiple comparisons: *p < .05, ** < .005, ***p < .002.

Based on the significant beneficial effects of CBD on neuroinflammation markers described above, we analyzed microglial morphology in the cortex of Veh‐treated *Arx* ^(GCG)7/Y^ or of CBD‐treated *Arx* ^(GCG)7/Y^ and age‐matched male controls. Numerous in vivo studies have demonstrated that CBD reduces neuroinflammation by suppressing microglial activation, thereby aiding in the restoration of brain homeostasis.^41^ Across multiple epileptic conditions, the activation of microglia is marked by observable changes in their morphology and functional properties.^42^ Building on these considerations, we analyzed the morphological complexity of microglia in the cortex of symptomatic *Arx* ^(GCG)7/Y^ mice, at the end of the Veh and CBD treatments and in untreated controls. For this analysis, we performed anti‐Iba1 immunostaining on cortices of young male mice (Figure 3A,B), followed by Sholl analysis examining microglial morphology and branching patterns (Figure 3C–I, Figure S4A–H). When compared to the XYWT profile, Veh‐treated *Arx* ^(GCG)7/Y^ microglia exhibited a Sholl intersection profile characterized by a significant increase in the number of intersections near the soma, followed by a marked decrease distally from the soma (Figure 3E). Furthermore, Veh‐treated *Arx* ^(GCG)7/Y^ microglia displayed a significant increase in the number of primary processes, along with a notable decrease in the average length of primary processes, maximum branch length, and convex hull area (Figure 3F–I). These morphological changes were significantly reversed by CBD, returning to those observed in the control microglia (Figure 3E–I). No significant differences were observed in the cell density, soma size, number of secondary processes, total process length, or average secondary process length of Iba1+ cells between *Arx* ^(GCG)7/Y^ and control mice, nor did CBD treatment affects these measures (Figure S4I). We therefore conclude that CBD reverses microglial morphology, which is likely influenced by an underlying inflammatory response, and in doing so, may contribute to mitigating the epileptic phenotype in this DEE murine model.

### 3.6 CBD reverses the defective isoform splicing switches of neurexin 1 and neurexin 3

**FIGURE 4:**
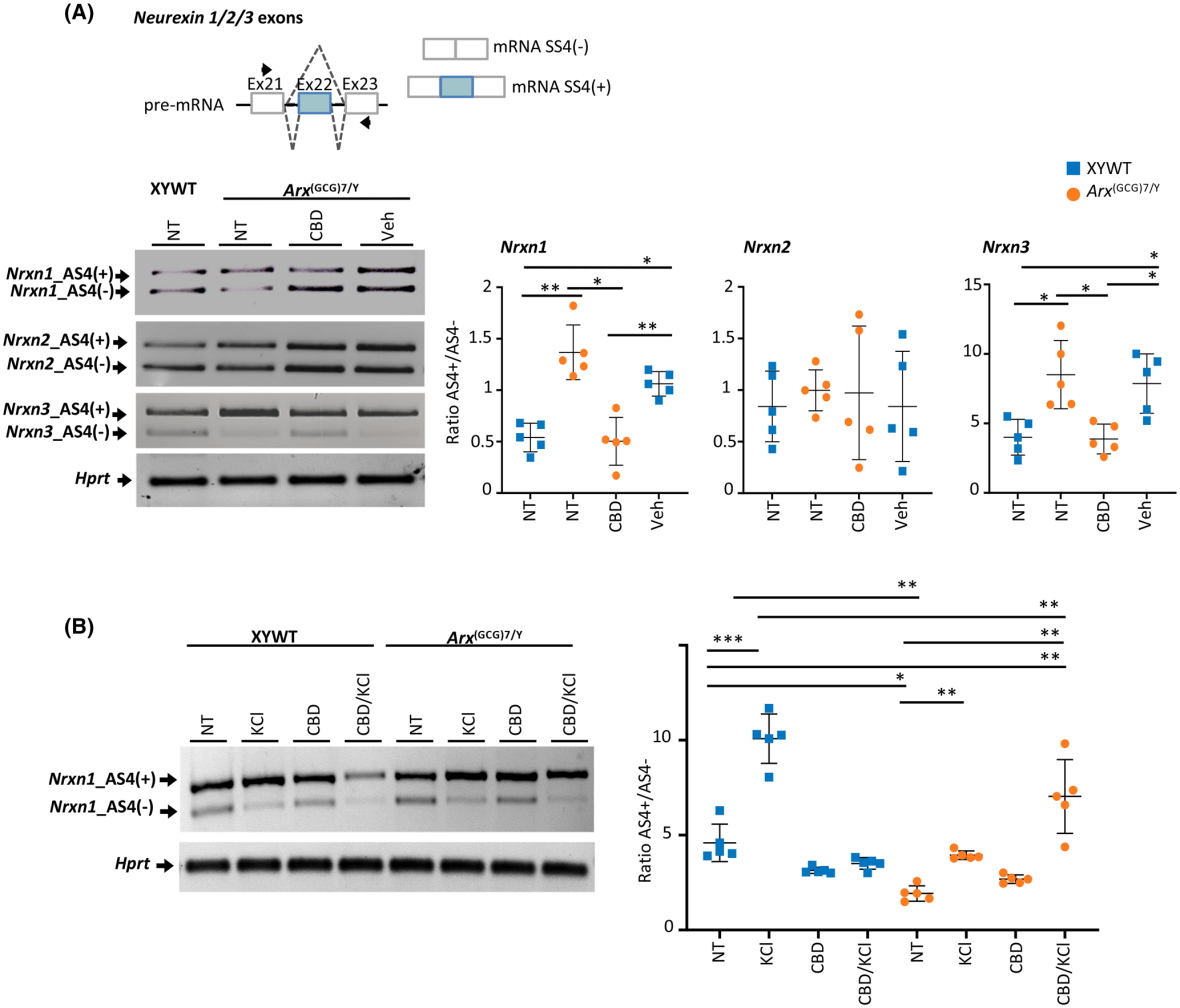
Impact of cannabidiol (CBD) on neurexin isoform splicing switches in *Arx* ^(GCG)7/Y^ mice. (A) Semiquantitative real‐time polymerase chain reaction (RT‐PCR) of alternative isoforms of *Nrxn*1, *Nrxn*2, and *Nrxn*3 AS4 in cortex isolated from XYWT and *Arx* ^(GCG)7/Y^ mice. Schematic representation of exon 22 splicing in murine *Nrxn1/2/3* genes is shown. Arrows indicate the positions of oligonucleotides used in transcript analysis. Ratio of band quantification of AS4(+)/AS4(−) in each experimental group is plotted. Dot plots represent mean ± SD of five independent mice. Two‐way analysis of variance (ANOVA) with Tukey multiple comparison test: *p < .05, ** < .005. (B) Semiquantitative RT‐PCR of alternative isoforms of *Nrxn*1 AS4 in resting, KCl‐depolarized cortical neurons, CBD treated‐cortical neurons, and CBD treated/KCl‐depolarized cortical neurons isolated from XYWT and *Arx* ^(GCG)7/Y^ pups. Ratio of band quantification of AS 4(+)/AS 4(−) is plotted. Dot plots represent mean ± SD of five independent experiments. Two‐way ANOVA with Tukey multiple comparison test: *p < .05, **p < .005, ***p < .001. NT, no treatment; Veh, vehicle.

In addition to its effects on neuroinflammation and microglia, CBD may rapidly modulate neuroplasticity by promoting neural network remodeling and potentially restoring normal synaptic function.^23^, ^43^ Therefore, we investigated whether CBD might counteract the abnormal neuroplasticity features associated with aberrant splicing switches of neurexin 1 (*Nrxn1*) and neurexin 3 (*Nrxn3*), which we previously detected in the *Arx* mutant cortex.^12^ Splicing of neurexin exon 22 produces two presynaptic NRXN isoforms—AS4(+) and AS4(−)—that differ in their interactions with the canonical postsynaptic neuroligin receptors in excitatory neurons, thereby influencing synaptic remodeling.^44^ Importantly, *Nrxn1* and *Nrxn2*, along with the *Nrxn3*, belong to the neurexin gene family, which has been linked to autism spectrum disorder (ASD), and epilepsy.^45^ Using a semiquantitative PCR assay, we analyzed the levels of AS4(+) and AS4(−) isoforms of *Nrxn1*, *Nrxn2*, and *Nrxn3* in the cortex isolated from *Arx* ^(GCG)7/Y^ young mice treated by daily intraperitoneal administration with CBD (100 mg/kg, *n* = 5) or Veh (*n* = 5) for 7 days, at the same age as the behavioral study (PND35–37; Figure 1A). Consistent with our previous findings in neonatal animals,^12^ we observed that the cortex of symptomatic untreated and Veh‐treated DEE1 mice exhibits a significant increase in the *Nrxn1* AS4(+)/AS4(−) and *Nrxn3* AS4(+)/AS4(−) ratios compared to mouse controls, whereas the *Nrxn2* AS4(+)/AS4(−) ratio remained unchanged (Figure 4A). Considering the distinct binding affinities of NRXN AS4(+) and AS4(−) isoforms for neuroligin receptors, this altered splicing ratio could compromise the appropriate neurexin–neuroligin interaction at the synaptic cleft, leading to a synaptopathy. Upon treatment, we observed that CBD is capable to rectify the altered neurexin repertoire for both *Nrxn1* and *Nrxn3* genes with a drastic decrease of AS4(+)/AS4(−) ratio in the cortex isolated from symptomatic *Arx* ^(GCG)7/Y^ mice (Figure 4A).

Further confirmation of the beneficial activity of CBD on the neurexin repertoire was found in *Arx* ^(GCG)7/Y^ depolarized cortical neurons. As previously reported, high K+ depolarization of the neuronal membrane alters synaptic plasticity by increasing the *Nrxn1* AS4(+)/AS4(−) ratio.^12^, ^45^, ^46^ By using the cortex of mutant embryos and age‐matched male controls, cortical neuronal cultures were generated (Figure S5A). First, by immunofluorescence with anti‐VGLUT1 and anti‐γ‐aminobutyric acid (GABA), we established that primary cultures of both genotypes had comparable proportions of glutamatergic and GABAergic neurons (respectively 70%:30%; Figure S5B,C). Successively, we carried out single treatment with CBD (10 μmol·L^−1^) or KCl (51 mmol·L^−1^), or with CBD and KCl in two sequential steps (CBD/KCl, 10 μmol·L^−1^ and 51 mmol·L^−1^, respectively; Figure S5D). Supporting the role of KCl in inducing changes in synaptic plasticity, increased transcript levels of *c*‐*fos* were found in KCl‐treated XYWT and KCl‐treated mutant primary neurons compared to the resting (untreated) neurons (Figure S5D). Regarding the *Nrxn1* AS4(+)/AS4(−) ratio, a significant increase was observed in KCl‐treated XYWT neurons and CBD/KCl‐treated mutant neurons (Figure 4B). Additionally, we observed that the response in CBD/KCl‐treated mutant neurons closely mirrored KCl‐treated XYWT neurons rather than KCl‐treated *Arx* ^(GCG)7/Y^ neurons, suggesting a synergistic effect of CBD and KCl in promoting a more appropriate synaptic plasticity response, potentially through the restoration of proper neurexin–neuroligin receptor clustering. In summary, these findings underscore the potential of neurexin 1 and neurexin 3 splicing switches as targets for CBD action. Given the involvement of these genes in various forms of ASD and pediatric epilepsy, this represents a significant advancement in exploring the applicability of CBD to other NDDs.

### 3.7 CBD reduces synaptic transmission in cultured primary neurons

**FIGURE 5:**
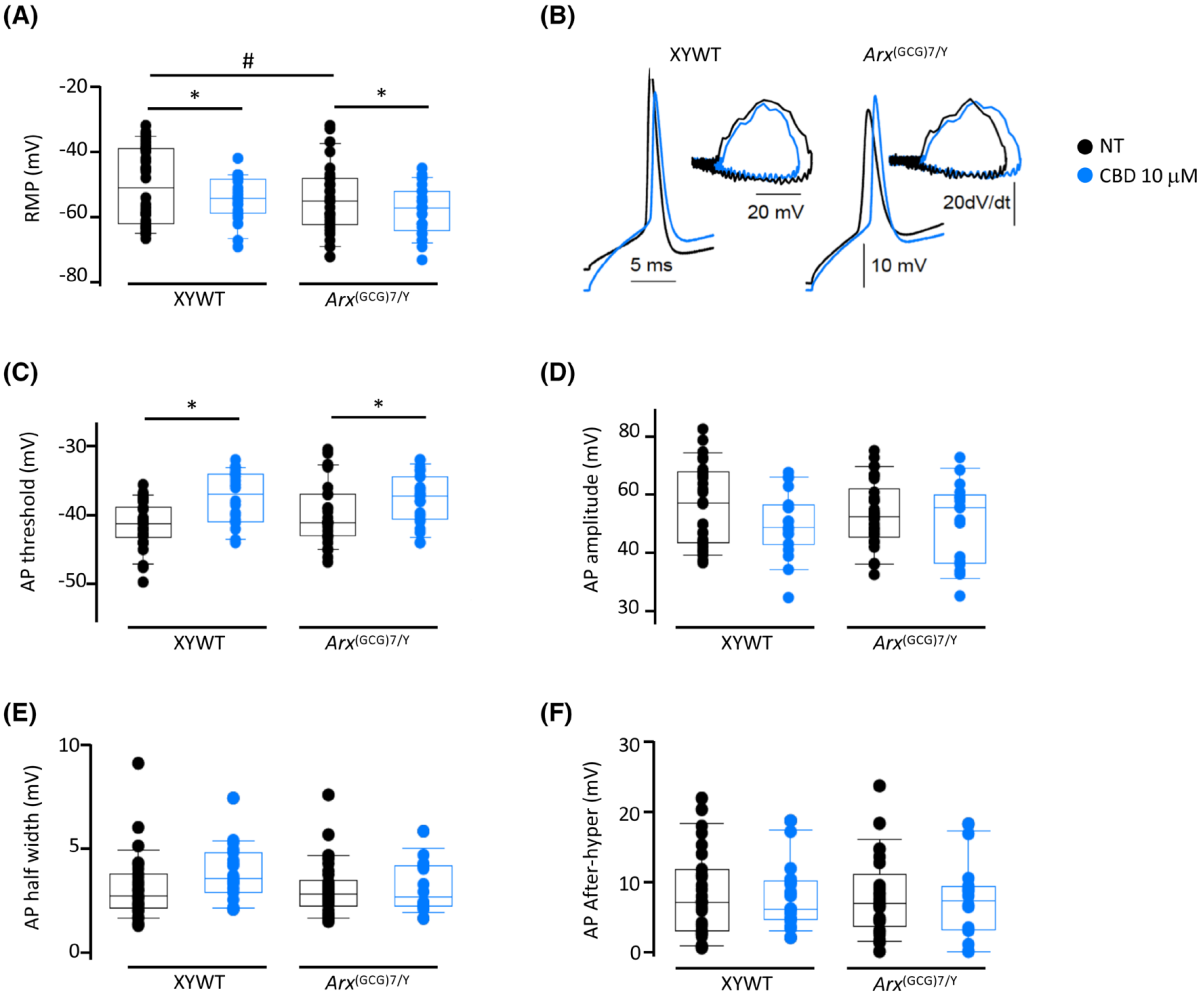
Cannabidiol (CBD) modulates membrane potential and action potential (AP) properties in cultured neurons. (A) Boxplots representing resting membrane potentials (RMP) recorded from XYWT neurons in control condition (black circles, 25 cells) and after CBD pretreatment (blue circles, 15 cells) and *Arx* ^(GCG)7/Y^ neurons in control condition (black circles, 23 cells) and after CBD (blue circles, 15 cells). (B) Superimposed AP traces recorded in XYWT (upper left) and *Arx* ^(GCG)7/Y^ (upper right) neurons in the control condition (black lines) and after CBD pretreatment (blue lines) and their corresponding phase–plane plot (bottom). (C) AP threshold measured from the same neurons as in panel A. (D) Boxplots representing AP amplitude values, with the same neurons as in panel A. (E) Boxplots representing AP half‐width values, with the same neurons as in panel A. (F) Boxplots representing AP afterhyperpolarization (After‐hyper) values, with the same neurons as in panel A. Two‐Way analysis of variance with Holm–Sidak multiple comparisons test, not treated (NT) versus CBD‐treated: *p < .05; NT XYWT versus not treated *Arx* ^(GCG)7/Y^ #*p* < .05.

Given the significant beneficial effects of CBD on synaptic plasticity response in *Arx* ^(GCG)7/Y^ primary neurons, we next assessed whether CBD could also exert a favorable effect on the intrinsic excitability of mutant neurons. We then performed in vitro patch‐clamp recordings on cortical neurons from days 13–14 after initiating a primary culture using the cortex of mutant embryos and age‐matched male controls.^47^ Pretreatments with 10 μmol·L^−1^ CBD were done on day 12 (for 2 h) in XYWT and *Arx* ^(GCG)7/Y^ neurons (Figure S5E). In 25 XYWT control neurons, we found a mean resting membrane potential (RMP) of −48 ± 2 mV, which decreased to −53 ± 2 mV in 23 *Arx* ^(GCG)7/Y^ cells (*p* = .042; Figure 5A). In pretreated neuronal cultures, CBD hyperpolarized both XYWT and *Arx* ^(GCG)7/Y^ neurons, lowering the RMP to −55 ± 2 mV and to −58 ± 2 mV, respectively (15 and 15 cells, respectively; two‐way analysis of variance, *p* = .036; Figure 5A). In the same cells, we elicited depolarizing steps (20 ms) to evoke a single action potential (AP; Figure 5B). Pretreatment with CBD depolarized AP threshold in both XYWT (from −41 ± 1 mV to −37 ± 1 mV) and *Arx* ^(GCG)7/Y^ neurons (from −40 ± 1 mV to −37 ± 1 mV; *p* = .019; Figure 5B,C). We analyzed other AP kinetic parameters such as amplitude (Figure 5D), half‐width (Figure 5E), and afterhyperpolarization (Figure 5F), and no differences between XYWT, *Arx* ^(GCG)7/Y^, and CBD treatments were observed.

**FIGURE 6:**
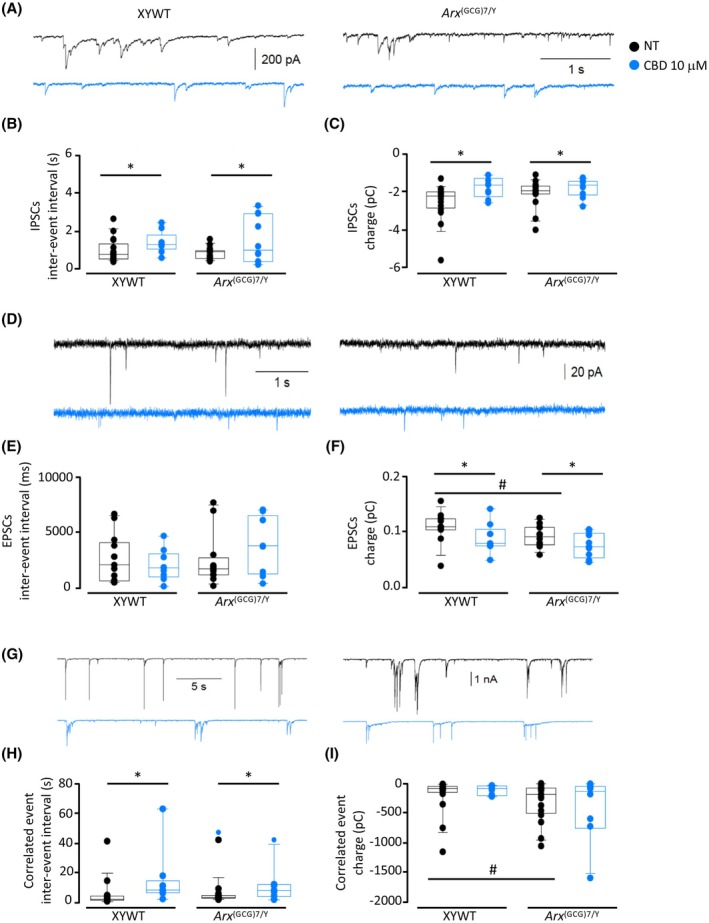
Cannabidiol (CBD) reduces synaptic transmission in XYWT and *Arx* ^(GCG)7/Y^ cultured neurons. (A) Typical traces showing individual inhibitory postsynaptic currents (IPSCs) recorded from XYWT (left) and *Arx* ^(GCG)7/Y^ (right) neurons in control condition (black traces) and after CBD (blue traces). (B, C) Boxplots showing the interevent interval and the mean charge event of individual events recorded from XYWT and *Arx* ^(GCG)7/Y^ neurons (17, 9, 17, and 10 cells, respectively). (D) typical traces showing individual excitatory postsynaptic currents (EPSCs) recorded from XYWT (*left*) and *Arx* ^(GCG)7/Y^ neurons in control (black traces) and after CBD pretreatment (blue traces). (E, F) Boxplots showing the EPSC interevent interval and the mean charge obtained from XYWT and *Arx* ^(GCG)7/Y^ cells (13, 10, 12, and 9 cells, respectively). (G) Typical traces recorded from XYWT (left) and *Arx* ^(GCG)7/Y^ (right) neurons and presented with enlarged scale to show the large correlated synaptic events, in control condition (upper) and after CBD (bottom). (H) Interevent interval of correlated events measured in XYWT and *Arx* ^(GCG)7/Y^ neurons (same cells as in panels B and C). (I) Boxplots representing the mean charge values of correlated synaptic events. Two‐way analysis of variance with Holm–Sidak multiple comparisons test, not treated (NT) versus CBD‐treated: *p < .05; NT XYWT versus NT *Arx* ^(GCG)7/Y^ #*p* < .05.

Spontaneous inhibitory postsynaptic currents (IPSCs) were recorded, in control condition or after CBD pretreatment, in XYWT neurons (17 and 9 cells, respectively) and in *Arx* ^(GCG)7/Y^ neurons (17 and 10 cells, respectively; Figure 6A–C). In control condition, no difference was observed in IPSC kinetic parameters measured in both genotypes. Pretreatment with CBD significantly reduced the frequency and the mean charge of IPSCs in both XYWT and *Arx* ^(GCG)7/Y^ neurons (*p* = .007 and *p* = .019, respectively; Figure 6B,C). The frequency of glutamatergic excitatory postsynaptic currents (EPSCs) was similar both in XYWT and *Arx* ^(GCG)7/Y^ neurons, whereas the EPSC mean charge was smaller in the mutated neurons (*p* = .042; Figure 6D,E). Moreover, CBD significantly reduced the EPSC mean charge in both XYWT and *Arx* ^(GCG)7/Y^ neurons (Figure 6F; *p* = .018). In both XYWT and *Arx* ^(GCG)7/Y^ neurons, we also identified multiple correlated synaptic events (correlated events; Figure 6G–I) due to the summation of several unitary postsynaptic currents. Although the frequency of correlated events was similar for XYWT and *Arx* ^(GCG)7/Y^ neurons (Figure 6H), *Arx* ^(GCG)7/Y^ neurons exhibited a significantly larger mean charge associated with these synaptic events (*p* = .037; Figure 6I), indicating a higher synchronization of synaptic activity in mutated neurons. Even for correlated events, CBD treatment was able to reduce the frequency in both genotypes (*p* = .049; Figure 6G,H), confirming the ability of CBD to decrease neuronal signaling in both conditions.

## 4 DISCUSSION

We demonstrate for the first time that the administration of CBD improves epileptic phenotype and extends the survival of *Arx* ^(GCG)7/Y^ mice. To our knowledge, CBD is the only drug to date that exerts such effect in this severe DEE mouse model. Remarkably, the *Arx* ^(GCG)7/Y^ replicates one of the most frequently reported polyalanine tract expansion mutations in ARX detected in DEE1/ISSX1 patients, making it a highly suitable system for evaluating promising novel treatments such as CBD.^4^, ^6^, ^12^, ^19^ Although unraveling the molecular components disrupted by the abnormal transcriptional activity of ARX is highly complex, we showed that CBD has a pleiotropic modifying action targeting simultaneously a range of unanticipated molecular and cellular targets affected in *Arx*‐DEE1 cortex, such as inflammation, microglia phenotype, synaptic function, and neuronal excitability.

### 4.1 CBD attenuates epileptic phenotype and increases survival

Our overall findings—by using video monitoring and a scoring matrix—show that CBD administration in young symptomatic DEE1 mice (PND35–37 to PND42–44) effectively ameliorates the epileptic phenotype. This was evidenced by a marked reduction in the frequency of SRS and a decrease in seizure burden, with these beneficial effects persisting for up to 7 days after the treatment was discontinued. A long‐term efficacy effect of CBD neuroprotective action has also been observed in preclinical rodent and piglet models of neurodegenerative diseases and newborn hypoxic–ischemic encephalopathy.^48^, ^49^ Mechanistically, these enduring effects might be caused by modifications in synaptic plasticity leading to lasting changes in global network connectivity that could continue even after the treatment has concluded.

As previously reported, patients with ARX polyalanine elongations have a significantly increased risk of premature death compared to healthy individuals.^7^ The major causes of death in these patients were not accurately described, but a respiratory illness or sudden unexpected death in epilepsy are reported as frequent death causes in DEEs.^50^ Remarkably, 30%–38% of *Arx* ^(GCG)7/Y^ mice die before weaning, and the remaining animals succumb within 3 months.^17^, ^19^ In our study, we showed that CBD significantly extends the lifespan of these mice—a notable finding, especially because other promising drugs like E2 have failed to prolong survival in this model.^17^ Because there were no differences in body weight between CBD‐treated and Veh‐treated mice, the increased survival is unlikely to be due to enhanced feeding. We therefore speculate that the extension in survival could be primarily driven by the potent anticonvulsant and disease‐modifying effects of CBD. This vital effect was also observed in murine models of DS and Leigh syndrome (LS), both of which exhibit markedly reduced lifespans in patients and animal models.^51^, ^52^ Importantly, because CBD treatment in *Arx* ^(GCG)7/Y^ mice commenced shortly after seizure onset, our findings strongly indicate that early CBD administration in DEE1/ISSX1 patients could serve as a highly effective therapeutic strategy, both suppressing seizures and reducing premature death risk.

In addition, the CBD dose used in this study is consistent with the effective anticonvulsant doses used in other mouse models of pharmacoresistant epilepsies, including DS, LS, and Angelman syndrome.^51^, ^52^, ^53^

### 4.2 CBD suppresses inflammatory markers

This study is the first to show that in the cortex of *Arx* ^(GCG)7/Y^ mice, the abnormal expression of a subset of molecules involved in neuroinflammatory response is reversed by CBD treatment. As secondary disease determinants arising from the *Arx* polyalanine elongation, these molecules could be considered specific targets for the development of targeted therapies for DEE1. We showed that CBD counteracts the transcriptional deficiency of *Pparg* and suppresses the upregulation of the proinflammatory genes *Mmp9* and *Ptgs2*. Given that *Pparg* is a positive transcriptional target of ARX^32^ and its protein product acts as a receptor for CBD,^23^ the stimulation of the *Pparg*–*Mmp9*–*Ptgs2* transcriptional axis could be one of the functional mechanisms through which CBD impacts on the ARX transcriptional program. Furthermore, we found that CBD reduces the elevated levels of the brain inflammatory marker TRPV1, along with its phosphorylated form, in the cortex of *Arx* ^(GCG)7/Y^ animals. Because TRPV1 levels increase in response to inflammatory signals,^54^ the excessive TRPV1 levels detected in the mutant cortex further support the notion that a proinflammatory state may contribute to the physiopathology of *Arx* ^(GCG)7/Y^ mice. However, the precise mechanism by which CBD reduces TRPV1—and consequently its phosphorylated form—remains unclear. We speculate that CBD may act on TRPV1 through a double mechanism (1) by downregulating TRPV1 protein via the *Pparg*–*Ptgs2* transcriptional axis contributing to offsetting the inflammation state and (2) by the desensitization of pTRPV1 channels—as observed in other epileptic seizure systems^24^, ^55^—thus dampening the abnormal neuronal excitability. Notably, we also demonstrated that anti‐inflammatory effects of CBD extend to other key inflammasome mediators that are overexpressed in the *Arx* ^(GCG)7/Y^ cortex. We showed that CBD significantly reduces the transcript levels of the proinflammatory immune biomarkers IL‐12, CD68, CCL2, and IRF3, found previously upregulated in rodent models of induced epilepsy and in pediatric patients with encephalopathy.^37^, ^56^ The finding that these CBD‐responsive molecules elicit an immune‐mediated inflammatory response in both murine models and clinical epilepsy cases further confirms that the mutant mouse cortex is distinctly marked by a proinflammatory state. The lack of effects in suppressing the activation of the proinflammatory genes *Tlr4*, *Irak4*, and *Myd88*—which are key innate immune molecules implicated in epileptogenesis^56^—highlights a selective anti‐inflammatory action of CBD in this DEE1 model. This discovery points to a previously underestimated role of inflammation in the *Arx* ^(GCG)7/Y^ mouse pathogenesis and underscores the potential of CBD anti‐inflammatory activity in preventing or reducing the severity of DEE1 epilepsy. Future omics studies will help clarify how CBD affects the transcriptional program driven by *Arx* polyalanine mutations, leading to the identification of primary and secondary disease determinants that are responsive to CBD.

### 4.3 CBD suppresses microglia morphology changes

Our findings reveal that as a corollary of its anti‐inflammatory activity, CBD offsets a defective morphological profile of microglia cells including shortened primary processes, increased intersections, and an overall reduction in branch length and convex hull area. Microglia are highly dynamic cells that continuously extend and retract their processes to survey the tissue microenvironment. As observed in in vivo and in vitro epilepsy models, microglial cells exposed to inflammatory proepileptogenic stimuli become activated, adopting a more amoeboid shape and displaying features similar to those seen in the *Arx* ^(GCG)7/Y^ cortex.^41^, ^42^ CBD has been shown to reduce microglial activation and lower proinflammatory marker levels in various neurological disease systems, including seizure models.^41^ On the other hand, the CBD‐responsive targets found deregulated in the mutant cortex—PPARγ, TRPV1, IL‐12, CD68, CCL2, and IRF3—are all mechanistically connected to microglial activation.^23^, ^54^, ^55^ We therefore speculate that the spontaneous epileptic activity seen in *Arx*‐DEE1 mice could lead to changes in the microglial state, and that CBD helps to restore microglial morphology by exerting its anti‐inflammatory effects. However, due to the complexity and variability of microglial phenotypes across different functional states, and because the effects on cellular motility are often model‐specific and not easily generalized, the understanding of the precise role of microglia in DEE1 mice is still in its early stages.

### 4.4 CBD suppresses neurexin‐splicing switch abnormalities and changes innate firing properties

Beyond its anti‐inflammatory effects, we uncovered that CBD exerts a neuroprotective activity in *Arx* ^(GCG)7/Y^ cortex and primary neurons. CBD corrects the aberrant splicing switches of neurexin 1 and 3 that we previously identified in the *Arx* mutant neocortex, contributing to the restoration of the molecular composition of the presynaptic membrane and the proper binding of neuroligins to the postsynaptic membrane as well.^12^ Given that the neuroligin–neurexin complex has been shown to selectively regulate interactions at both glutamatergic and GABAergic synapses,^57^, ^58^ we propose that CBD may potentially restore the disrupted balance between excitatory and inhibitory neurotransmission. Consistently with these data, we found a synergistic activity of CBD with KCl at ameliorating *Nrxn1* AS4(+)/AS4(−) ratio and thus the depolarization response in primary mutant neurons.

Emerging evidence suggests that CBD may influence splicing mechanisms, although the exact mechanisms remain under investigation. CBD has been shown to directly bind to EFTUD2, a component of the spliceosome complex that processes precursor mRNAs to produce mature mRNAs, indicating a potential role in spliceosome regulation.^59^ Regarding the direct interactions between CBD and neurexins, or with neuroligin–neurexin complex, no data have been reported to date. This is a highly intriguing field of research due to the association of neurexin genes with ASD^45^ and the potential therapeutic applications of CBD in these disorders.^60^

Remarkably, in a previous study, an unexpected role for neurexins in the endocannabinoid‐dependent regulation of neural circuits was reported. Presynaptic neurexin splice variants containing the SS4 segment modulate excitatory synaptic strength by regulating the postsynaptic biosynthesis of the endocannabinoid 2‐arachidonoylglycerol, indicating a link between the neurexin family and endocannabinoid signaling.^61^ Given the complexity of the overlapping pathways regulated by ARX, including alternative splicing control and synaptic plasticity,^12^ further research is needed to understand how CBD affects alternative splicing switches on a global scale and to pinpoint its precise effects on neurexin genes. Consistent with previous studies,^62^ we further showed that, in both cultured control and mutant primary neurons, CBD decreases excitability by inducing hyperpolarization and increasing the action potential threshold. This neuroprotective activity of CBD mirrors its ability to influence the intrinsic properties of depolarized neurons, thus dampening hyperexcitability and thereby counteracting recurrent seizures.^60^ In line with this, we showed that CBD reduces the frequency, the mean charge of IPSCs, and the mean charge of EPSCs in both genotypes. The question of how CBD dampens hyperexcitability in these primary neurons may be answered by the simultaneous interactions of CBD with multiple targets, some of them analyzed in this study.

In summary, this study adds *Arx* ^(GCG)7/Y^ to the range of epilepsy murine models in which CBD treatment is effective. Given the rarity of DEE1 caused by polyalanine elongation mutations in *ARX*, our findings—obtained using a mouse model that replicates key aspects of the epileptic phenotype—encourage further research that may ultimately lead to clinical assessment of CBD in DEE1 for ameliorating seizure control and survival. This could lead to the development of novel, targeted treatments that address both the genetic and inflammatory aspects of this rare form of DEE exploring the applicability in other ARX‐related disorders. Further studies are needed to better understand how CBD affects ARX‐driven disease mechanisms and its ability to regulate microglia–neuron interactions and neuroligin–neurexin signaling.

## AUTHOR CONTRIBUTIONS

Lucia Verrillo and Denise Drongitis contributed to animal behavior experiments and cellular studies and data analysis. Loredana Poeta and Adriano Barra contributed to data acquisition of animal experiments. Katiuscia Martinello and Sergio Fucile contributed to data acquisition and analysis of electrophysiology recording. Fabio Arturo Iannotti and Vincenzo Di Marzo contributed to data acquisition and analysis of CBD treatments. Gaetano Terrone contributed to data analysis of epilepsy behavior. Fabio Arturo Iannotti, Vincenzo Di Marzo, and Maria Giuseppina Miano contributed to the experimental design. Maria Giuseppina Miano contributed to conceptualization, supervision, and writing—review and editing. All authors critically revised the article, commented on the drafts of the manuscript, and approved the final version.

## CONFLICT OF INTEREST STATEMENT

G.T. has received speaker honoraria from Jazz Pharmaceuticals, UCB, and Neuraxpharm. None of the other authors has any conflict of interest to disclose. We confirm that we have read the Journal's position on issues involved in ethical publication and affirm that this report is consistent with those guidelines.

## Supporting information


Appendix S1.

Movie S1.

Video S1.

## Data Availability

The data that support the findings of this study are available from the corresponding author upon reasonable request.
