# Supplementary material for: Cannabidiol attenuates epileptic phenotype and increases survival in a mouse model of developmental and epileptic encephalopathy type 1
Source: Epilepsia. 2025 Jul 3;66(10):4035–52. doi: 10.1111/epi.18522 (PMC12605674; doi:10.1111/epi.18522)
Supplement: Supplementary file 1 — Appendix S1. [file EPI-66-4035-s002.pdf]

Supporting information

Materials and methods

Mouse welfare and ethical statements. All experiments in the mice were conducted at Animal house Facility of Institute of Genetics and Biophysics "Adriano Buzzati-Traverso" in accordance with the principles of a European Communities Council Directive (86/609/EEC, 2010/63/EU). All procedures were approved by the Italian Ministry of Health (D.L.gs n. 26/2014) under the accreditation n°307/2018-PR E58D.8 and n°555/2022-PR E58D.26 in accordance with the Institutional Animal Care guidelines of the Institute of Genetics and Biophysics "Adriano Buzzati-Traverso".

Mouse model of Arx-DEE1. *Arx*$^{(GCG)7/Y}$ knockin colony was purchased from the RIKEN BioResource Center in Japan [strain name: *Arx*(GCG)7-1 KI (B6), RBRC03654]. Mice were maintained by crossing heterozygous females *Arx*$^{(GCG)7/X}$ with WT males C57BL/6J. Male adult C57BL/6 mice (weight, 20–25g; age, 6–9 weeks) were obtained from Charles River Laboratories. Genotyping of *Arx*$^{(GCG)7/Y}$ was performed according to the protocol of the RIKEN BioResource Center.[1] All animals were maintained in room with a 12h dark:light cycle at 20-24°C and humidity of 45±10 %, with *ad libitum* access to food and water throughout the study period. *Arx*$^{(GCG)7/X}$ heterozygous female pregnancy was assessed after overnight mating and embryonic age was calculated as E0.5 when the vaginal plug was detected. Mice were sacrificed following deep anesthesia with ketamina and all efforts were made to minimize discomfort and pain to animals. All *Arx*$^{(GCG)7/Y}$ mice develop recurrent spontaneous seizures between P25-P30.[1] Since *Arx* is located on the X chromosome, only *Arx*$^{(GCG)7/Y}$ males exhibit the DEE1 phenotype, while *Arx*$^{(GCG)7/X}$ females remain asymptomatic[1].

in-vivo treatments. Animals were randomized either the CBD or vehicle group. Animal identification codes were chosen at random by one investigator, and then an independent investigator assigned the animal to receive either CBD or vehicle. Plant-derived-highly purified CBD (batch no RCA1741) was supplied by GW Research Ltd., Cambridge, UK (now part of Jazz Pharmaceuticals). *Arx*$^{(GCG)7/Y}$ animals in treatment group received one daily intraperitoneal (i.p.) injection of 100mg/kg of CBD, as used in other epilepsy-murine models.[2] CBD was dissolved in ethanol and administered intra peritoneally at 100 mg $\cdot$ Kg^{-1} in a vehicle solution (ethanol:Tween 80:NaCl 0.9% 1:1:18) beginning on day 8 after quantification of baseline seizure frequency. Control animals received one daily i.p. injection of vehicle (1:1:18) to confirm absence of anticonvulsant activity.

Video recording and epilepsy monitoring. *Arx*$^{(GCG)7/Y}$ animals were video monitored 24 hours a day for three weeks throughout the entire experimental period. CCTV cameras (C2Cube Ezviz) were

connected to PC with video footage recorded using PhyLink software.Video footages were obtained during the light and night phase from the initial 7 days (*baseline* phase), the followed 7 days (*treatment* phase) and the final 7 days (*washout* phase) were coded offline to analyze the mouse behavior. Two blinded independent researchers were trained to identify and code convulsive behaviors using a modified Racine scale.[3] Six stages – summarized in Table S1- were considered for behavioral seizure quantification: Score 0/Stage-1, absence-like immobility (no change in behaviour); Score 1/Stage-2, sudden behavioural arrest, straight tail, facial grooming; Score 2/Stage-3, head nodding, straight tail, increased facial grooming; Score 3/Stage-4, forelimb clonus with lordotic posture; Score 4/Stage-5, forelimb clonus with rearing and falling; Score 5/Stage-6, generalized tonic-clonic activity with loss of postural tone, wild jumping and running followed by generalized convulsions, often resulting in death. A movie clip showing *Arx*$^{(GCG)7/Y}$ stages from 3 to 5 is available in Supplementary materials (Movie S1). The mutant animals were confirmed as epileptic by visual observation (Racine scale ≥3), as previously described. [3] Only animals classified as epileptic within 4-5 weeks from the birth were used for the analysis of seizures to minimize age-related variability. Scores of ≥ 3 were recorded and included in the results as these reflected clearly identifiable tonic-clonic convulsion. Number, duration and severity of seizure were evaluated. The seizure burden was calculated from the sum of all multiplied scores of severities and the corresponding frequencies during the observation period, by applying the following formula: (number of graded-3 seizures x 3) + (number of graded-4 seizures x 4) + (number of graded-5 seizures x 5), as reported elsewhere.[4] The seizure burden ratio was calculated from the seizure burden by using the following formula: mean seizure burden in *baseline* interval/mean seizure burden in *washout* interval, as reported elsewhere.[4] No disease manifestations were observed in carrier females

Assessment of survival. *Arx*$^{(GCG)7/Y}$ mice were monitored daily to assess survival. Animals found dead was assumed to have died as a consequence of either one seizure or continuous seizure activity.

Immunohistochemistry and microglia morphometric analysis. Whole mice brains were post-fixed in PFA 4% for 12 h at 4°C, and paraffin-embedded for microtome sectioning (10 μm coronal sections). For immunofluorescent labeling, sections were pre-treated for antigen unmasking by boiling for 25 minutes in pH 6 citrate buffer. Sections were blocked in 0.5% Triton X-100 and 10% Normal goat serum (Blocking solution), for 1 h at room temperature (RT) and incubated ON at RT with primary antibodies, rabbit anti-Iba1 (Wako, 019-19741; 1:1000), mouse anti-NeuN (Millipore MAB377; 1:500). The following day, they were incubated with secondary antibodies raised against mouse or rabbit IgG generated in goat and coupled to either Alexa Fluor 488 or Alexa Fluor 594 (Life technologies; 1:400). Nuclei were counterstained with DAPI (10μg/mL, Life Technologies).

Z-stacks confocal images (0.65 μm step size) of cortical Iba1+ microglia cells were acquired with an LSM 900 Zeiss confocal microscope under 20X and 40X oil objective, at 1024x1024 pixels resolution. Manual morphological analysis was performed on maximum intensity projection RGB images with FIJI Image J software plugin Simple Neurite Tracer (SNT), setting the radius step size at 3μM.

Primary neuron isolation, ex-vivo treatments and immunocytochemistry. Cortical neurons were prepared from $Arx^{(GCG)7/Y}$ and XY WT neonatal brains (3 mice *per* culture experiment). Cortices were dissected and dissociated to single cells suspension with 0.125% trypsin. Cells were maintained in Neurobasal medium (NB, Gibco), 1% B-27 plus supplement (Gibco), 10% Horse serum (Gibco), 2 mM L-glutamine and penicillin/streptomycin (Gibco). At *in vitro* cultured day 4 (DIV4), cells were treated with 10mM Cytosine arabinoside (Sigma-Aldrich) for 48 hours. Membrane depolarization was induced at DIV11 by addition of depolarization buffer with high potassium concentration (170 mM KCl, 2 mM $CaCl_2$, 1 mM $MgCl_2$, 10 mM Hepes) for 10 min, by adding 30% depolarization buffer, 30% conditioned Neurobasal medium, 40% fresh Neurobasal medium (KCl final concentration = 51 μM). NaCl buffer solution was used as a control.[5,6] Neurons were pre-incubated with CBD 10 μM for 1 hour at 37°C before depolarization induction. For immunocytochemistry studies, WT and $Arx^{(GCG)7/Y}$ primary neurons were grown on coverslips and then fixed in 4% paraformaldehyde (Sigma) 1X phosphate buffered saline (PBS). The fixed samples were washed, permeabilized in 0.5% Triton X-100 1X PBS, blocked in 5% bovine serum albumin (Sigma), and incubated over night at 4°C with the primary antibodies rabbit anti-GABA (A2052, Sigma-Aldrich, 1:100), anti-VGLUT1 (sc-377425, Santa Cruz Biotechnology, 1:400). The following day, they were incubated with secondary antibodies raised against mouse or rabbit IgG generated in goat and coupled to either Alexa Fluor 488 or Alexa Fluor 594 (Life technologies; 1:400). Images were taken randomly under a NIKON confocal microscope and processed by ImageJ software. Immunoreactive areas were quantified by Threshold and Analyse particles plug-in.

RNA extraction, quantitative Real Time PCR and alternative splicing analysis. Total RNA extraction and DNAse treatment were performed according to the manufacturer's protocols (Life Technologies). Reverse transcription was performed with Superscript III Reverse Transcription kit (Life Technologies) and Quantitative PCR was executed with SYBR-Green-based reagents (Bio-Rad) by using a CFX96 real-time PCR Detection system (Bio-Rad). Alternative splicing abundances were determined using the AmpliTaq DNA Polymerase (Applied Biosystems) on the Biorad PCR System (Applied Biosystems). The oligonucleotide sequences used are reported in Table S2. The measures of transcript analysis were normalized to *18S* and *Hprt* RNA level. Each experiment assay

was performed in triplicate in three independent experiments. DNA fragment intensities were quantified by using ImageJ software.

Western blotting analysis. Cortices were homogenized in lysis solution (1× TNE Buffer, 1% (v/v); TritonX-100, plus 1% protease inhibitor cocktail) at pH 7.4. Lysates were kept in an orbital shaker incubator at 220 × g, at 4 °C for 30 min, and then centrifuged for 15 min at 13,000 × g at 4 °C. The supernatants were transferred to clear tubes and quantified by the DC Protein Assay (5000111, Bio-Rad). Samples (60 µg of total protein) were boiled for 5 min in Laemmli SDS loading buffer and loaded on 8–10% SDS-polyacrylamide gel electrophoresis and then transferred to a PVDF membrane. Filters were incubated overnight at 4 °C with rabbit anti-TRPV1 (SAB3501027, Sigma-Aldrich, 1:500); polyclonal rabbit anti-phospo-TRPV1 (pTRPV1, Ser502) (PA5-64860, Thermo Fisher, 1:500). The mouse monoclonal anti-tubulin (T8203, Sigma-Aldrich, 1:1000) was used to check for equal protein loading. Reactive bands were detected by chemiluminescence by using Clarity Western ECL (1705060, Bio-Rad). Images were analyzed on a Chemi-Doc station with Quantity-one software (Bio-Rad).

Electrophysiology for in vitro recordings. Patch-clamp recordings were made on DIV12 cultured cortical neurons prepared from *Arx*$^{(GCG)7/Y}$ and XY WT neonatal brains. Postsynaptic currents were recorded with a Multiclamp 700B amplifier and Clampex 10.5 softwareat -70 mV holding potential in a normal external solution (NES).[7] Excitatory post-synaptic currents (EPSCs) were recorded in the presence of bicuculline (20 µM). NES had the following composition (in mM): 140 NaCl, 10 Hepes, 2.8 KCl, 2 $CaCl_2$, 2 $MgCl_2$, and 10 Glucose; pH 7.3 adjusted with NaOH. Patch pipettes were filled with intracellular solution containing (mM):140 KCl, 10 Hepes, 2 $MgCl_2$, 2 MgATP, and 0.5 EGTA; pH 7.2 adjusted with KOH. CBD was applied to cells for 2 hours the day before the experiments and then washed out. The analysis of synaptic events was performed with Clampfit 11 software (Axon Instruments) using detection sliding templates. The detection criterion was calculated from the template-scaling factor and from how closely the scaled templates fit the different recorded events. The mean event charge of a correlated or individual synaptic event was measured as the time integral of the GABAergic or glutamatergic synaptic currents. Resting membrane potential (RMP) values were obtained as soon as the whole-cell configuration was reached. No cell was excluded based on RMP value, to detect possible depolarizing effect due to genotype and/or treatment.

The threshold of single action potentials elicited by 40 ms current steps from -70 mV was measured by differentiating the spike voltage to time (dV/dt). This was plotted against the voltage to create a phase plane plot. The threshold was defined as the voltage at the point of deflection for dV/dt to be greater than zero. Only action potentials surpassing 0 mV were included in the study.

Statistical analysis. Statistical analysis was performed using GraphPad Prism 7 software. In behavioral experiments, normal distribution of data was analyzed by Kolmogorov-Smirnov Test. Data for median seizure counts were normally distributed, while data for duration of seizure were not normally distributed, therefore Repeated Measures One-Way ANOVA for number of seizure and Kruskal-Wallis test with multiple comparisons for duration of seizures were used. The D'Agostino & Pearson test and One-Way Repeated measures ANOVA were used for seizure burden analysis. The Mann Whitney test was used for seizure burden ratio analysis. Survival analyses were conducted using Kaplan–Meier analysis with Mantel-Cox log rank test; mice who died before weaning (P28) were excluded from this analysis. In RT- PCR, western blotting experiments, and morphological analysis One-way and Two-Way ANOVA were conducted. In all experiments the significance threshold was set at $p<0.05$. For electrophysiological experiments, statistical comparisons between groups were made with Two-Way ANOVA followed by pairwise multiple comparisons with the Holm-Sidak method. The power of all performed tests was >0.8 ($\alpha = .05$). $p < 0.05$ was taken as significant.

Web Resources

NCBI, https://www.ncbi.nlm.nih.gov/
UCSC, https://genome.ucsc.edu/
OMIM, https://www.omim.org/
mVISTA, https://genome.lbl.gov/vista/mvista/submit.shtml

References

1. Kitamura K, Itou Y, Yanazawa M, et al. Three human ARX mutations cause the lissencephaly-like and mental retardation with epilepsy-like pleiotropic phenotypes in mice. Hum Mol Genet. 2009;18:3708-3724.
2. Kaplan JS, Stella N, Catterall WA, Westenbroek RE. Cannabidiol attenuates seizures and social deficits in a mouse model of Dravet syndrome. Proc Natl Acad Sci U S A. 2017; 114:11229-11234.
3. Ihara Y, Tomonoh Y, Deshimaru M, Zhang B, Uchida T, Ishii A, Hirose S. Retigabine, a Kv7.2/Kv7.3-Channel Opener, Attenuates Drug-Induced Seizures in Knock-In Mice Harboring Kcnq2 Mutations. PLoS One. 2016;11:e0150095.
4. Patra PH, Barker-Haliski M, White HS, et al. Cannabidiol reduces seizures and associated behavioral comorbidities in a range of animal seizure and epilepsy models. Epilepsia. 2019; 60:303-314.
5. Ding X, Liu S, Tian M, et al. Activity-induced histone modifications govern Neurexin-1 mRNA splicing and memory preservation. Nat Neurosci. 2017;20:690-699.
6. Drongitis D, Caterino M, Verrillo L, Santonicola P, Costanzo M, Poeta L, Attianese B, Barra A, Terrone G, Lioi MB, Paladino S, Di Schiavi E, Costa V, Ruoppolo M, Miano MG. Deregulation of microtubule organization and RNA metabolism in Arx models for lissencephaly and developmental epileptic encephalopathy. Hum Mol Genet. 2022; 31:1884-1908.
7. Martinello K, Sciaccaluga M, Morace R, Mascia A, Arcella A, Esposito V, Fucile S. Loss of constitutive functional γ-aminobutyric acid type A-B receptor crosstalk in layer 5 pyramidal neurons of human epileptic temporal cortex. Epilepsia. 2018; 59:449-459.

8. Zhu Y, Qi C, Korenberg JR, Chen XN, Noya D, Rao MS, Reddy JK. Structural organization of mouse peroxisome proliferator-activated receptor gamma (mPPAR gamma) gene: alternative promoter use and different splicing yield two mPPAR gamma isoforms. Proc Natl Acad Sci USA. 1995; 92:7921-5.

Table S1. Racine scale used to classify the behavioural seizure phenotype in *Arx*$^{(GCG)7/Y}$ mice[3]

Score	Behavioral expression
**0**	No change in behaviour
**1**	Sudden behavioural arrest, straight tail, facial grooming/washing
**2**	Head nodding, straight tail, increased facial grooming/washing
**3**	Forelimb clonus with lordotic posture
**4**	Forelimb clonus with rearing and falling
**5**	Generalized tonic-clonic activity with loss of postural tone, wild jumping, often resulting in death

Table S2. List of oligonucleotides used in this study.

Name	F* and R** sequence
Genotyping	
Arx WT°	F-AAAGGCGAAAAGGACGAGGAAAGG<br>R-CTTTAGTCCCCTTCCTGGCACAC
$Arx^{(GCG)7}$	F-AAAGGCGAAAAGGACGAGGAAAGG<br>R-TGTTCAATGGCCGATCCCAT
Sry	F-TGAATGCATTTATGGTGTGGTCCC<br>R-TGTATGTGATGGCATGTGGGTTCC
qRT-PCR	
Hprt	F- TTGGCTTTTCCAGTTTCACTAATG<br>R- TGCCCTTGACTATAATGAGTACTTCAG
18S	F- CAATGCGGCGGCGTTATTCCCAT<br>R- AATCTGTCAATCCTGTCCGT
c-fos	F- TCCACCCCAGAGTCTGAGGAGG<br>R- CTGAGCCACTGGGCCTAGAT
*Nrxn1*_AS4	F- TGTTGGGACAGATGACATCGCC<br>R- GAGAGCTGGCCCTGGAAGGG
*Nrxn2*_AS4	F- GTGCGCTTTACTCGAAGTGGTG<br>R- CCCATTGTAGTAGAGGCCGGAC
*Nrxn3*_AS4	F- TTGTGCGCTTCACCAGGAATG<br>R- AGAGCCCAGAGAGTTGACCTTG
Il12	F- GGACCAAACCAGCACATTGA<br>R- AGGCACAGGGTCATCATCAA
Il1β	F- TGACGGACCCCAAAAGATGA<br>R- TCTCCACAGCCACAATGAGT
Il16	F- CGCTATGAAGTTCCTCTCTGC<br>R- CAGGTCTGTTGGGAGTGGTA
Il10	F- ATAACTGCACCCACTTCCCA<br>R- GCATTAAGGAGTCGGTTAGCA
Tnfα	F- GGTGCCTATGTCTCAGCCTCTT<br>R- GCCATAGAACTGATGAGAGGGAG
Cd68	F- AGGGTGGAAGAAAGGTAAAGC<br>R- AGAGCAGGTCAAGGTGAACAG
Ccl5	F- CTTTGCCTACCTCTCCCTCG<br>R- CGGTTCCTTCGAGTGACAAA
Ccl2	F- GAGTAGGCTGGAGAGCTACAAGAG<br>R- AGGTAGTGGATGCATTAGCTTCAG
Myd88	F- AACAAAGGAACTGGGAGGC<br>R- GTCTGTTCTAGTTGCCGGATC
Tlr4	F- AGATCTGAGCTTCAACCCCT<br>R- CATGCCATGCCTTGTCTTCA
Irak4	F- CTGTGGATGAAAACCGTGAAC<br>R- AGAGTACATTGCTTCCACCG
Irf3	F- GGCTGACTTTGGCATCTTCC<br>R- CGCAACACTTCTTTCCGGTT
Pparg	F- ACAGGCCGAGAAGGAGAAG<br>R- TCTTGCACGGCTTCTACGG
Mmp9	F- GGATCCCCAGAGCGTCATT<br>R- TTTGGAAACTCACACGCCAG
Ptgs2	F- GCAAATCCTTGCTGTTCCAACC<br>R- GGAGAAGGCTTCCCAGCTTTTG
Socs3	F- GATTTCGCTTCGGGACTAGC<br>R- GGAAACTTGCTGTGGGTGAC

*forward; ** reverse; ° Wild Type

Movie S1. Epilepsy-associated *Arx* $^{(GCG)7/Y}$ mice phenotype

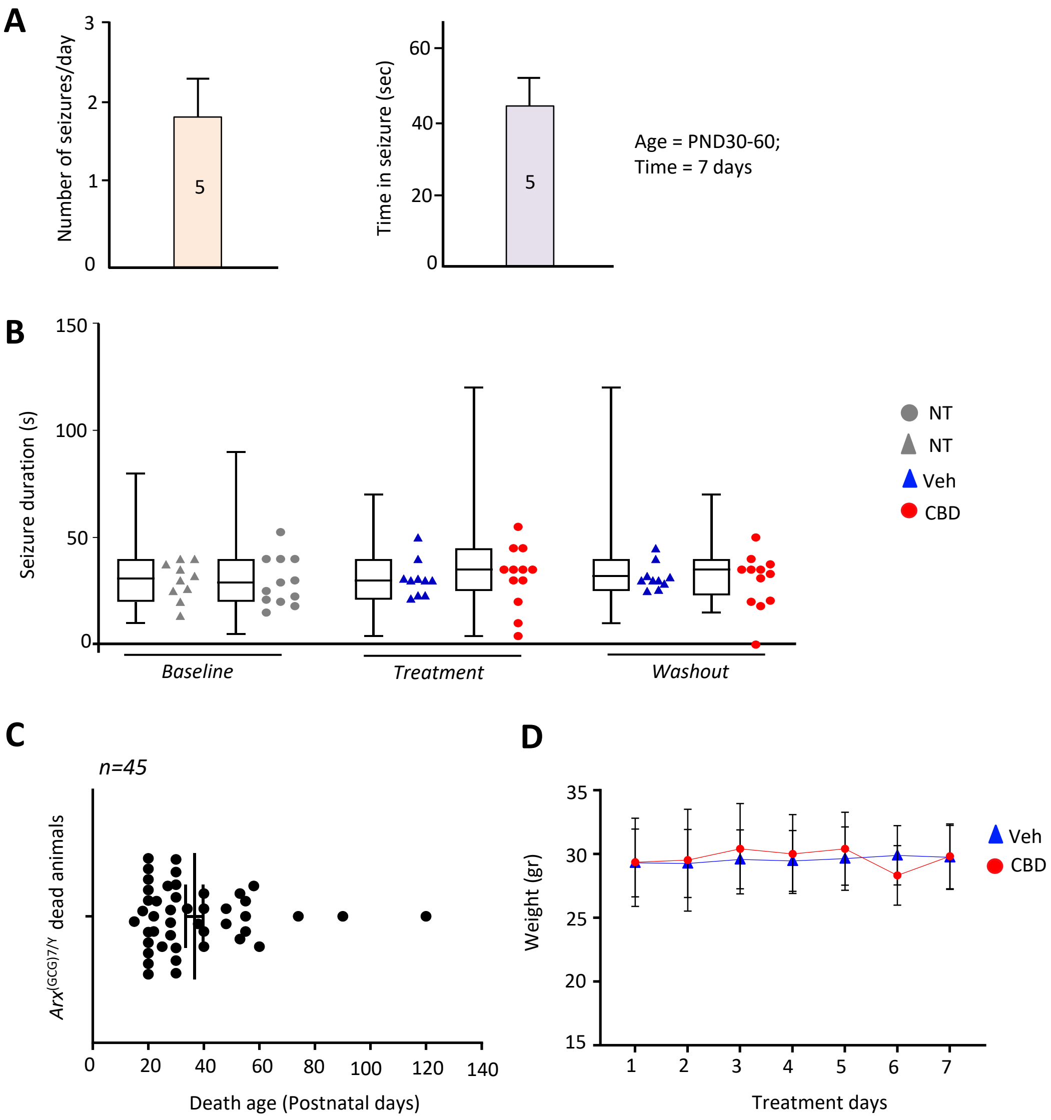

Figure S1. A. Mean number and duration of seizure per day in *Arx*$^{(GCG)7/Y}$ (n=5) mice; **B.** Box plots depicting the median seizure duration in the two experimental groups vehicle-treated (Veh n=10) and CBD-treated epileptic *Arx*$^{(GCG)7/Y}$ (CBD n=12) mice: during the *baseline* phase (days 1–7 days) before the *treatment* phase; during the CBD or Veh treatment phases (days 8–14 days); and during the *washout* phase (days 15–21). Individual data points plotted beside the boxes are the averaged data for individual mice during each phase of the experiment. Kruskal-Wallis with multiple comparisons was applied. **C.** Mortality data collected from *Arx*$^{(GCG)7/Y}$ mice: 38% of animals dies before weaning (PND28), while the majority of animals dies within 3 months. Data represent mean ± S.E.M., each dot represents a mouse, n=45; **D.** Weight measurements during the treatment phase in vehicle-treated (Veh n=10) and CBD-treated (CBD n= 12) animals.

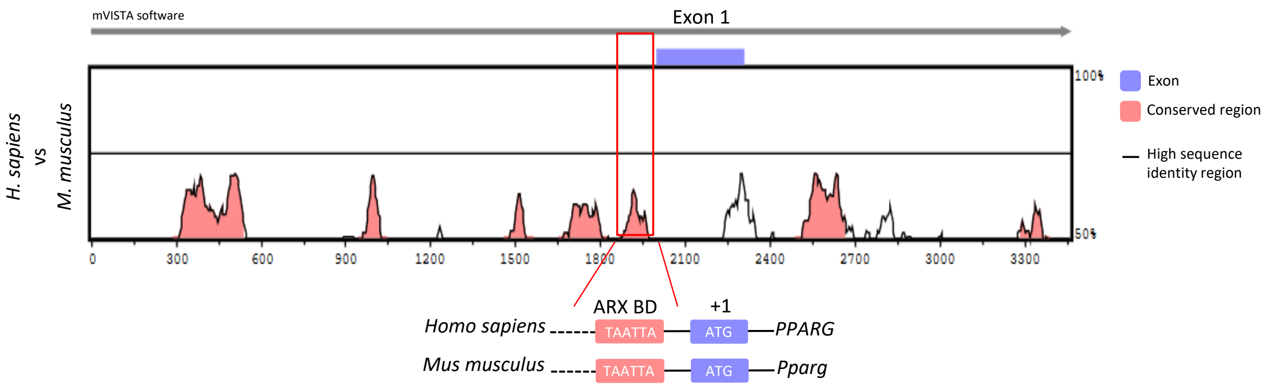

Figure S2. Alignment of the human genomic sequence spanning 2 kb upstream of exon 1 of human *PPARG* gene with the corresponding genomic region of murine *Pparg* gene including the sequence promoter previously identified[8] and annotated in Ensembl database (http://www.ensembl.org/ID ENSMUSR00000438633). The nucleotide sequences were downloaded from UCSC database: for Homo sapiens the coordinates used were Chr3: 12,285,368-12,287,367 (UCSC GRCh38) and for Mus musculus Chr6: 115,335,912-115,337,911 (UCSC GRCm39).The analysis was performed by mVISTA tool. The criteria used were a minimum of 65% nucleotide identity with a window size of over 100 bp. The positions of the ARX binding site (ARX BD, 5'-TAATTA-3') was numbered from ATG (+1) of both *PPARG/Pparg* genes: *H. sapiens* ARX BD (-1165/-1160 bp, Chr3 12,286,349 - 12,286,355) and *M. musculus* ARX BD (-970/-964 bp, Chr6 115,336,942 - 115,336,948).

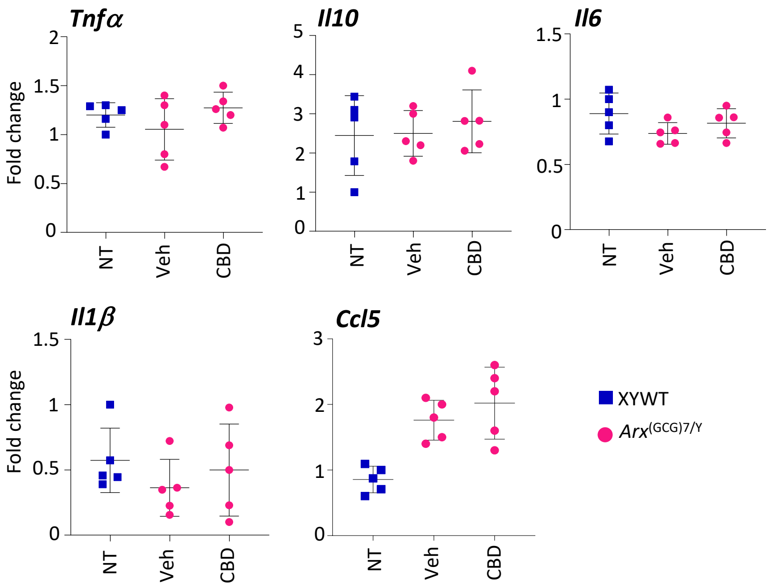

Figure S3. mRNA levels of *Tnfα, Il10, Il6, Il1β* and *Ccl5* in the cortex of XYWT, Veh-treated *Arx*$^{(GCG)7/Y}$ and CBD-treated *Arx*$^{(GCG)7/Y}$ mice measured by qRT-PCR. Transcript levels were quantified using the 2^-ΔΔCt method normalizing to 18S rRNA as the internal control (mean $\pm$ SEM of three replicates from 5 mice for experimental group). NT, not treatment; Veh, Vehicle treatment; CBD, CBD treatment. Differences were assessed using Two-way ANOVA with Tuckey's multiple comparisons.

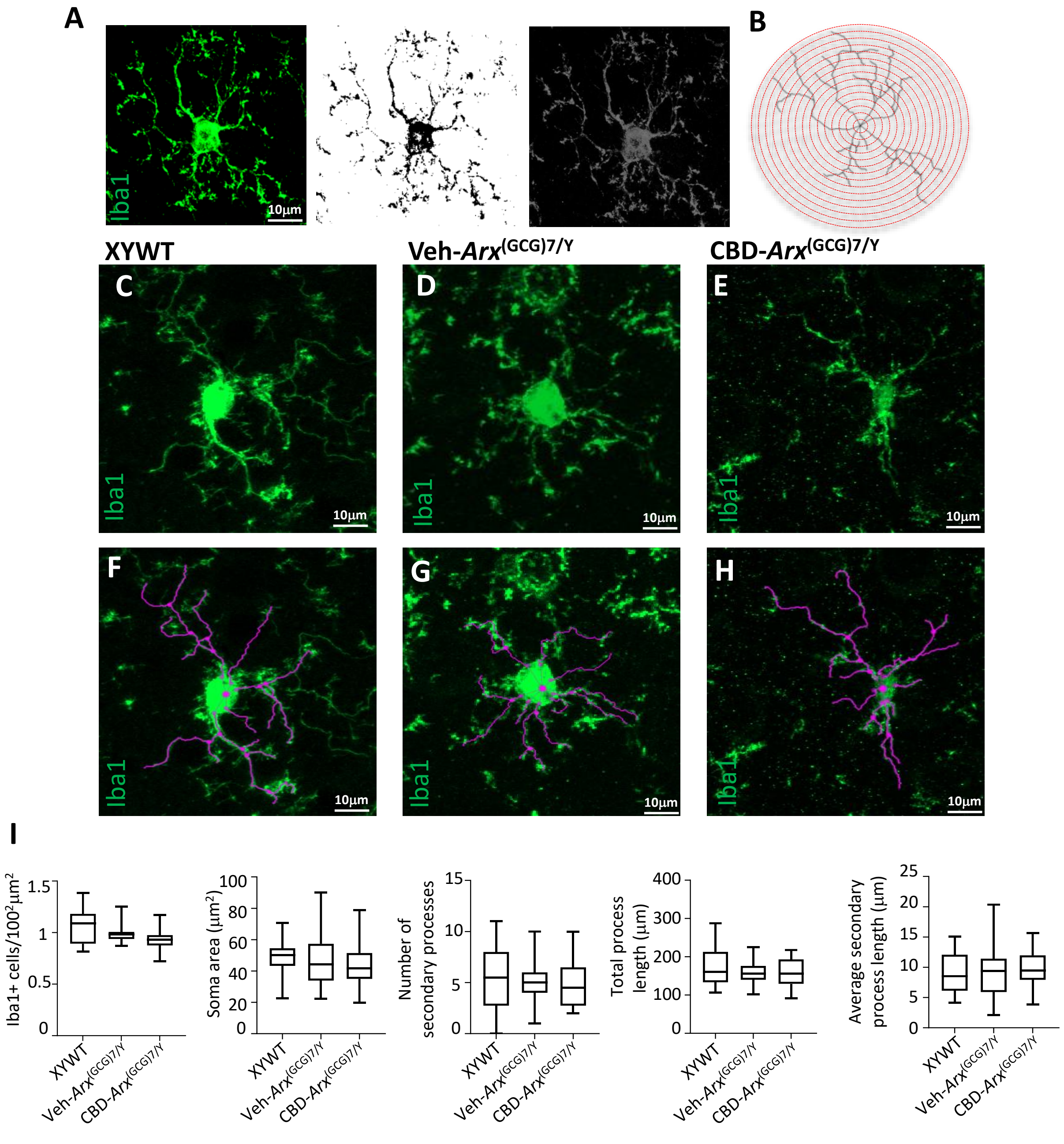

Figure S4. Analysis of microglia morphology in the cortex of XYWT, Veh-treated *Arx*$^{(GCG)7/Y}$ and CBD-treated *Arx*$^{(GCG)7/Y}$ mice. **A.** Schematic diagram of Sholl analysis. The results of Sholl analysis were processed using ImageJ. **B.** Skeletonized cell framework from (A) to be analyzed for morphological features. **C-E.** Confocal micrographs showing *Iba1*+ microglia in cortex.

F-H. Individual cells were subject to Sholl and skeleton analysis. **I.** Analysis of Iba1+ content(field analyzed n=16 XYWT; n=19 Veh-treated *Arx*$^{(GCG)7/Y}$; n=18 CBD-treated *Arx*$^{(GCG)7/Y}$), soma area (cells n=41 XYWT; n=46 Veh-treated *Arx*$^{(GCG)7/Y}$; n=42 CBD-treated *Arx*$^{(GCG)7/Y}$), number of secondary processes, total process length, and average secondary process length (cells n=18 XYWT; n=19 Veh-treated *Arx*$^{(GCG)7/Y}$; n=18 CBD-treated *Arx*$^{(GCG)7/Y}$). Differences were assessed using Two-way ANOVA with Tuckey's multiple comparisons.

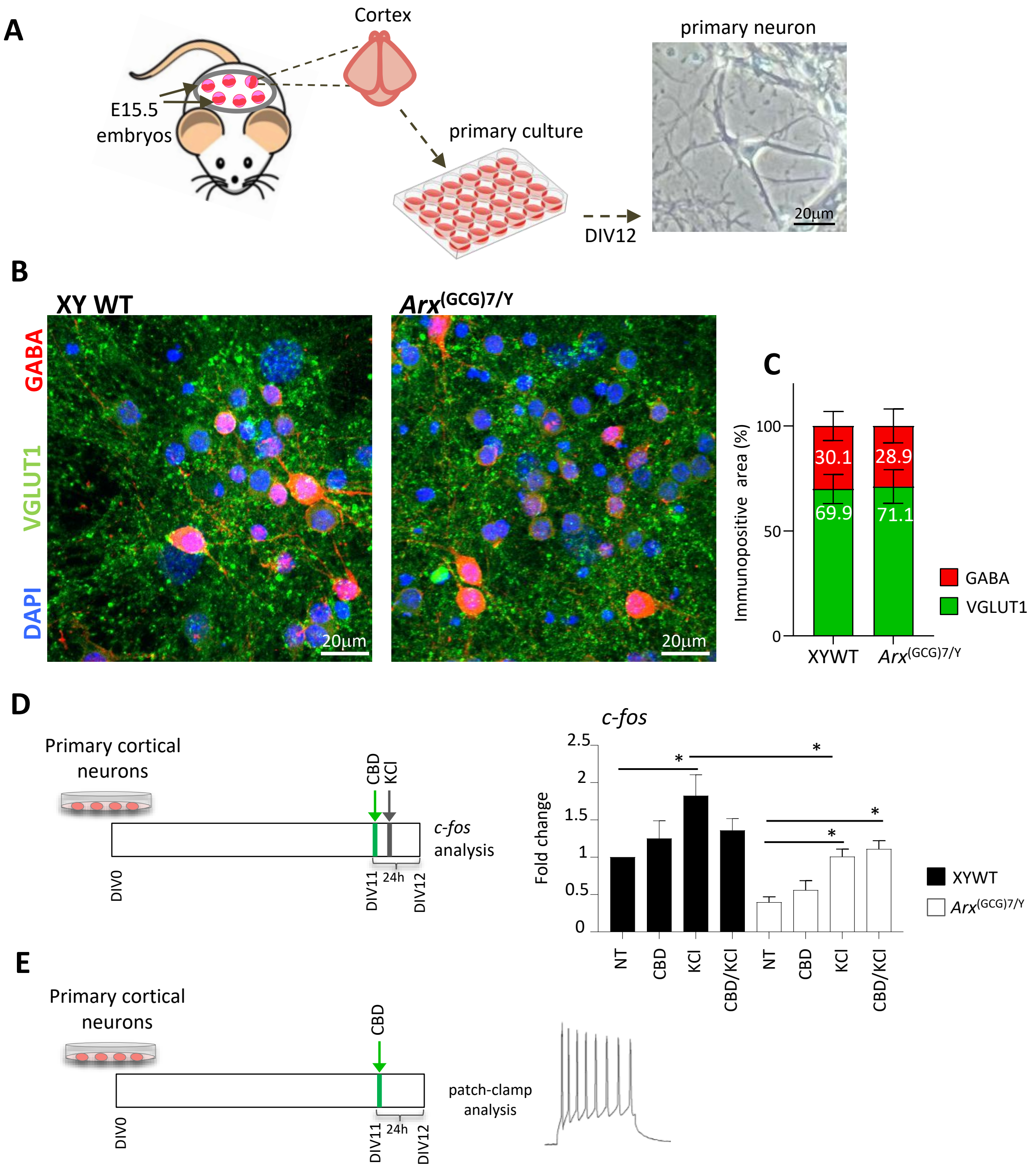

Figure S5. Analysis of primary cultured neurons isolated from XYWT and *Arx*$^{(GCG)7/Y}$ embryos.

A. Scheme of the experimental steps to generate primary cortical cultures; **B.** Immunofluorescent co-staining of GABA (*red*, GABAergic neurons) and VGLUT1 (*green*, Glutamatergic neurons) in neuron cultures (DIV12). Nuclei are labeled with DAPI (*blue*); **C.** Stacked bar graph showing the percentage of immunopositive area GABA+ and VGLUT1+ of primary neurons in XYWT and *Arx*$^{(GCG)7/Y}$ cultures; **D.** Analysis of *c-fos* expression in XYWT and *Arx*$^{(GCG)7/Y}$ primary neurons upon the following treatments: CBD [10μM] x 30 minutes, KCl [51mM] x 10 minutes and CBD/KCl (CBD 10μM x 30 minutes plus KCl 51mM x 10 minutes). *c-fos* transcript levels were quantified using the 2^-ΔΔCt method normalizing to 18S rRNA as the internal control (mean + SEM of three replicates for experiment). NT, untreated. The scheme of the treatment steps is showed. Differences were assessed using Two-way ANOVA with Tuckey's multiple comparisons, *p<0.05. **E.** Scheme of CBD [10μM] pre-treatment for patch-clamp analysis (for 2 hours 24 hours before recordings) in primary cortical XYWT and *Arx*$^{(GCG)7/Y}$ cultures.
