# Supplementary material for: Cannabidiol attenuates epileptic phenotype and increases survival in a mouse model of developmental and epileptic encephalopathy type 1
Source: Epilepsia. 2025 Jul 3;66(10):4035–52. doi: 10.1111/epi.18522 (PMC12605674; doi:10.1111/epi.18522)
Supplement: Supplementary file 3 — Video S1. [file EPI-66-4035-s003.docx]

Video S1

Representative video of behavioural seizure phenotype in *Arx*^(GCG)7/Y^ mouse. The recording captures spontaneous seizure activity progressing from stage 3 to stage 5 of the Racine scale. The mutant mouse initially shows head nodding, a straightened tail, and increased facial grooming (stage 3), followed by forelimb clonus accompanied by a lordotic posture, rearing, and falling (stage 4). The episode culminates in generalized tonic-clonic activity, characterized by loss of postural tone and wild jumping (stage 5). Continuous 24-hour video monitoring was conducted using a C2Cube Ezviz CCTV camera.
